# Exosomal circSHKBP1 promotes gastric cancer progression via regulating the miR-582-3p/HUR/VEGF axis and suppressing HSP90 degradation

**DOI:** 10.1186/s12943-020-01208-3

**Published:** 2020-06-29

**Authors:** Mengyan Xie, Tao Yu, Xinming Jing, Ling Ma, Yu Fan, Fengming Yang, Pei Ma, Huning Jiang, Xi Wu, Yongqian Shu, Tongpeng Xu

**Affiliations:** 1 Department of Oncology, The First Affiliated Hospital of Nanjing Medical University, Nanjing, China; 2 Department of Oncology, Sir Run Run Hospital, Nanjing Medical University, Nanjing, China; 3 Jiangsu Key Lab of Cancer Biomarkers, Prevention and Treatment, Collaborative Innovation Center for Cancer Personalized Medicine, Nanjing Medical University, Nanjing, China; 4 Department of Oncology, Zhenjiang First People’s Hospital, Zhenjiang, China

**Keywords:** CircSHKBP1, Gastric cancer, MiR-582-3p, HUR, VEGF, HSP90, Exosome

## Abstract

**Background:**

Circular RNAs (circRNAs) play important regulatory roles in the development of various cancers. However, biological functions and the underlying molecular mechanism of circRNAs in gastric cancer (GC) remain obscure.

**Methods:**

Differentially expressed circRNAs were identified by RNA sequencing. The biological functions of circSHKBP1 in GC were investigated by a series of in vitro and in vivo experiments. The expression of circSHKBP1 was evaluated using quantitative real-time PCR and RNA in situ hybridization, and the molecular mechanism of circSHKBP1 was demonstrated by western blot, RNA pulldown, RNA immunoprecipitation, luciferase assays and rescue experiments. Lastly, mouse xenograft and bioluminescence imaging were used to exam the clinical relevance of circSHKBP1 in vivo.

**Results:**

Increased expression of circSHKBP1(hsa_circ_0000936) was revealed in GC tissues and serum and was related to advanced TNM stage and poor survival. The level of exosomal circSHKBP1 significantly decreased after gastrectomy. Overexpression of circSHKBP1 promoted GC cell proliferation, migration, invasion and angiogenesis in vitro and in vivo, while suppression of circSHKBP1 plays the opposite role. Exosomes with upregulated circSHKBP1 promoted cocultured cells growth. Mechanistically, circSHKBP1 sponged miR-582-3p to increase HUR expression, enhancing VEGF mRNA stability. Moreover, circSHKBP1 directly bound to HSP90 and obstructed the interaction of STUB1 with HSP90, inhibiting the ubiquitination of HSP90, resulting in accelerated GC development in vitro and in vivo.

**Conclusion:**

Our findings demonstrate that exosomal circSHKBP1 regulates the miR-582-3p/HUR/VEGF pathway, suppresses HSP90 degradation, and promotes GC progression. circSHKBP1 is a promising circulating biomarker for GC diagnosis and prognosis and an exceptional candidate for further therapeutic exploration.

## Introduction

Gastric cancer (GC) is the fifth most frequently diagnosed cancer and the third leading cause of cancer death worldwide [1]. It threatens human health with an age-standardized five-year net survival rate ranging from 20 to 40% [2]. Therefore, investigating effective biomarkers for GC diagnosis and prognosis as well as targets for drug therapy exhibit great medical significance. Accumulated evidence has demonstrated the role of noncoding RNAs (ncRNAs) as epigenetic regulators in gastrointestinal malignancies [3, 4]. Circular RNAs (circRNAs) are a class of ncRNAs produced by back-splicing and their loop structure makes them more stable [5]. CircRNAs are highly abundant and specifically expressed in tissue, and thousands of them have differential expression between tumor and normal tissue [6, 7]. Individual circRNAs can act as microRNAs (miRNAs) sponges [8, 9]. MiRNAs post-transcriptionally silence target genes by binding to the 3′ untranslated region (3’UTR) of messenger RNA (mRNA) [10]. In addition to sponging miRNAs, circRNAs such as circFoxo3 and circPABPN1 can also function through interactions with proteins to induce cancer cell apoptosis, block cell cycle progression and inhibit cell proliferation [11–13]. It has been demonstrated that circRNAs are enriched and stable in exosomes. Exosomes are nanosized extracellular vesicles secreted by most cells, harboring abundant RNAs and proteins that can modulate the biological processes of cells and act as circulating biomarkers for various diseases. Studies show that serum exosomal circRNA can distinguish patients with cancer from healthy controls, and exosomal circRNAs can become biomarkers for liquid biopsy [14, 15].

Hu-antigen R (HUR), a member of the ELAV family, is one of the best known RNA binding proteins (RBPs) that selectively recognizes and binds to adenylate/uridylate-rich elements (AREs) [16]. AREs are adenylate uridylate rich regions in 3′ UTRs of mRNAs in mammalian cells and they mediate rapid degradation of mRNAs [17]. HUR post-transcriptionally modulates its target genes by stabilizing their mRNAs, and it is involved in cell growth and tumorigenesis [18–20].

Another popular oncoprotein is heat shock protein 90 (HSP90), an evolutionarily conserved molecular chaperone that is involved in stabilizing and activating proteins in response to stress [21]. HSP90 is overexpressed in tumors compared to normal tissues, and its function as a buffer for genetic lesions allows mutant proteins to retain their functions while permitting cancer cells to tolerate the imbalanced microenvironment [22].

In this study, we showed that circSHKBP1 was overexpressed both in tumors and serum exosomes of GC patients, and it was correlated with advanced pathological staging and poor survival. CircSHKBP1 promoted GC progression in vivo and in vitro by sponging miR-582-3p and decoying HSP90. Our study provided a new insight into the pathogenesis of GC.

## Materials and methods

### Patient samples

A total of 224 GC cases who had surgically proven primary GC and received a D2 radical gastrectomy (R0 resection) were obtained from Surgery Department of First Affiliated Hospital of Nanjing Medical University. Specimens were collected in accordance with institutional protocols. Tumors and adjacent normal tissues of 8 cases were used for RNA sequencing. Blood samples of 20 GC patients and 20 health controls (with no diagnosed cancer) were collected. Tumors and paired normal tissues of 72 cases were frozen tissues. Other samples of 152 cases were embedded with paraffin to make the tissue microarray, and clinicopathological features, which included age, sex, tumor site, tumor size, differentiation grade, Lauren classification, TNM stage (American Joint Committee on Cancer classification, AJCC), vascular invasion, lymphatic invasion and neural invasion, were shown in Table 1. This study was approved by the Medical Ethics Committee of First Affiliated Hospital of Nanjing Medical University. Written informed consent was obtained from all participants.

**Table 1:** Relationship between circSHKBP1 expression and clinicopathologic factors of patients with gastric cancer

Parameter	No. of patients	circSHKBP1(low)	circSHKBP1(high)	*P* value (* *P* <0.05)
Sex				
Male	108	51	57	0.283
Female	44	25	19	
Age (year)				0.224
< 60	49	28	21	
≥ 60	103	48	55	
Tumor size (cm)				0.033*
< 5	65	39	26	
≥ 5	87	37	50	
Tumor site				0.320
Upper	16	7	9	
Middle	58	32	26	
Lower	64	33	31	
Diffuse	14	4	10	
Differentiation grade				0.001*
Well-moderate	60	40	20	
Poor-undifferentiation	92	36	56	
Lauren classification				0.037*
Intestinal	72	41	31	
Diffuse	64	25	39	
Mixed	16	10	6	
T stage				0.052
T1-T2	34	22	12	
T3-T4	118	54	64	
Lymph node status				0.194
Negative	39	23	16	
Positive	113	53	60	
Distant metastasis				0.513
M0	142	72	70	
M1	10	4	6	
TNM stage				0.027*
I-II	53	33	20	
III-IV	99	43	56	
Vascular invasion				0.036*
Negative	104	58	46	
Positive	48	18	30	
Lymphatic invasion				0.827
Negative	127	64	63	
Positive	25	12	13	
Nervous invasion				1.000
Negative	138	69	69	
Positive	14	7	7	

The TNM Staging System is based on the tumor (T), the extent of spread to the lymph nodes (N), and the presence of metastasis (M)

### Cell culture

The AGS, HUVECs cell lines were purchased from the American Type Culture Collection (USA) and the HEK-293 T, HGC27, BGC823, MGC803, and GES1 cell lines were from Type Culture Collection of the Chinese Academy of Sciences (Shanghai, China). The HUVECs, HEK-293 T, HGC27, BGC823 and GES1 cells were cultured in RPMI 1640 medium (Gibco, Carlsbad, CA, USA). The MGC803 cells were cultured in Dulbecco’s modified Eagle’s medium (DMEM) (Gibco). The AGS cells were cultured in F12K medium (Wisent, Canada). All the cell lines were supplemented with 100 μg/ml streptomycin, 100 U/ml penicillin and 10% fetal bovine serum (FBS) at 37 °C in a humidified atmosphere of 5% CO2. Transcription was blocked by the addition of 2 μg/ml actinomycin D (AAT Bioquest, CA, USA). Cycloheximide (CHX) (Sigma-Aldrich, MO, USA), MG132 (Selleck Chemicals, USA) and NMS-E973 (Selleck Chemicals) were used at the indicated concentrations.

### RNA preparation and quantitative real-time PCR (qRT-PCR)

Total RNA was extracted from the cells or tissues using the TRIzol reagent (Invitrogen, MA, USA). The nuclear and cytoplasmic fractions were extracted using PARIS™ Kit (Thermo Fisher, MA, USA). Isolated RNA was used for the reverse transcription reaction with HiScript Q RT SuperMix for qPCR (Vazyme, Jiangsu, China). Quantitative RT-PCR was carried out with SYBR Green PCR Master Mix (Vazyme) using an ABI Prism 7900 Sequence detection system (Applied Biosystems, Canada). GAPDH was used as an internal control, and the results for each sample were normalized to GAPDH expression. For RNase R treatment, 2 μg of total RNA was incubated for 20 min at 37 °C with or without 3 U/μg of RNase R (Epicentre Technologies, WI, USA) in 1× reaction buffer, and the resulting RNA was purified using RNeasy MinElute cleaning Kit (Qiagen, Valencia, CA) and then transcribed into cDNA. The primers are listed in Additional file 1.

### Plasmids and siRNA transfection and lentiviral transduction

The plasmid pcDNA3.1-CMV-circSHKBP1 was designed and synthesized by Hanbio Biotechnology (Shanghai, China). siRNAs targeting circSHKBP1 and miRNA mimics or inhibitors were designed and synthesized by RiboBio (Guangzhou, China). The plasmids and miRNA mimics or inhibitors were transfected into cells with Lipofectamine 3000 (Invitrogen). The siRNAs were transfected into the cells by DharmaFECT4 (Dharmacon, IL, USA). The lentivirus vector (pGLV3/GFP/Puro) containing shRNAs targeting circSHKBP1 and vector (pGLV5/GFP/Puro) overexpressing circSHKBP1 were generated by GenePharma (Shanghai, China), which were added to BGC823 cells. Stable cell lines were obtained by selection with puromycin. CMV-MCS-EF1α-luciferase-PGK-Blasticidin (Yijing Biotechnology, Nanjing, China) was then transfected into these cell lines for bioluminescence imaging. (sequences listed in Additional file 2).

### RNA sequencing (RNA-seq) analysis

Total RNA was isolated using TRIzol reagent and RNA quantification and quality was assured by NanoDrop 2000 (Thermo Fisher). RNA integrity and gDNA contamination test by denaturing agarose gel electrophoresis. RNA from each sample was subjected to the RiboMinus Eukaryote Kit (Qiagen) to remove ribosomal RNA prior to RNA-seq library construction. Sequencing library was determined by Agilent 2100 Bioanalyzer using the Agilent DNA 1000 chip kit (Agilent, CA, USA). The libraries were adjusted to 10 nM before cluster generation. The cDNA was then sequenced using a HiSeq 2000 system (Illumina, SanDiego, CA, USA) and a 100-bp paired-end run.

### RNA fluorescence in situ hybridization (FISH)

Cy3-labeled specific probe to circSHKBP1 and FAM-labeled specific probe to miR-582-3p were designed and synthesized by RiboBio and the signals was detected by the FISH Kit (RiboBio) according to the manufacturer’s instructions. Cells were grown to the exponential phase and were 40–50% confluent at the time of fixation. After permeabilization (1 × PBS/0.5% Triton X-100), the cells were hybridized in hybridization buffer with specific probes to circSHKBP1, U6 and 18S at 37 °C overnight. The hybridization buffer was then gradually washed off with 4× SSC (including 0.1% Tween-20), 2× SSC and 1× SSC at 42 °C. Nuclei were counterstained with 4,6-diamidino-2-phenylindole (DAPI) (RiboBio). Confocal images were captured using Zeiss AIM software and a Zeiss LSM 700 confocal microscope system (Carl Zeiss Jena, Oberkochen, Germany).

### Transwell assays

Transwell invasion assay and migration assay were performed in 24-well plates (Corning, MA, USA), using a 6.5-mm diameter Transwell chamber with 8-μm pore polycarbonate membrane insert (Corning). The bottom of upper chambers was coated with fibronectin (Merck Millipore, Darmstadt, Germany). After 48 h of transfection, BGC823 cells (3 × 10^4^) or HGC27 cells (2 × 10^4^) were plated on the upper chambers coated with or without 50 μl of Matrigel (Corning) in serum-free medium. RPMI 1640 containing 10% FBS was added to the lower chambers as a chemoattractant. After incubation for 12 h at 37 °C, cells were fixed with 4% paraformaldehyde, stained with crystal violet solution, and counted at × 200 magnification under a microscope. The assay was repeated three times in duplicate. The numbers of cells counted in five random fields were averaged.

### Cell counting kit-8 (CCK8) assay

After 48 h of transfection, BGC823 cells (3 × 10^3^) or HGC27 cells (2 × 10^3^) were seeded into 96-well plates (Corning). Then, 10 μl of CCK8 (Beyotime, Jiangsu, China) solution was added to each well at the appointed time. After 1 h of incubation at 37 °C, the absorbance at 450 nm was measured using an automatic microplate reader (Synergy4; BioTek, Winooski, VT, USA).

### 5-Ethynyl-2′-deoxyuridine (EdU) incorporation assay

EdU assays were performed with a Cell-Light EdU DNA Cell Proliferation Kit (RiboBio). Cells were seeded 30% confluent in 6-well plates after 48 h of transfection and were continuously cultured for 24 h. After incubation with 50 μM EdU for 2 h, the cells were fixed in 4% paraformaldehyde and stained with Apollo Dye Solution. Hoechst 33342 was used to stain the nucleic acids within the cells. Images were obtained with a Nikon Ti microscope (Nikon, Tokyo, Japan), and the number of EdU-positive cells was counted.

### Western blot, immunohistochemistry (IHC), immunofluorescence (IF) and immunoprecipitation (IP) assay

Proteins in cells and tissues were extracted with RIPA lysis buffer (Thermo Fisher). Serum proteins were extracted with Serum Protein Extraction Kit (Qcheng Bio, China). Western blot assays were performed according to details previously reported [23]. the immuno-complexes were detected with ECL Western Blotting Substrate (Thermo Fisher), visualized with BIO-RAD (BIO-RAD Gel Doc XR+, USA). The following antibodies were used (11000): anti-β-actin (Beyotime, AF0003); anti-α-tubulin (Beyotime, AF0001); anti-GAPDH (Beyotime, AF0006); anti-HSP90 (Proteintech, 60,318–1-Ig); anti-HUR (Proteintech, 11,910–1-AP); anti-EIF2S1 (Proteintech, 11,170–1-AP); anti-VEGF (Proteintech, 19,003–1-AP); anti-Calnexin (Abcam, ab92573); anti-CD63 (Abcam, ab134045); anti-TSG101 (Abcam, ab125011); anti-CD81 (Proteintech, 66,866–1-Ig); anti-STUB1 (Proteintech, 55,430–1-AP).

IHC, IF and IP was performed as previously described [24]. IHC was performed with antibodies against HUR and VEGF (Proteintech, 1:200). IF was performed with antibody against CD31 (Proteintech, 11,265–1-AP, 1:200). The images were scanned by Pannoramic SCAN (3DHistech, Hungary). IP was performed with anti-HSP90 antibody (Proteintech, 1:200) and appropriate control IgG (Merck Millipore), and the immunoprecipitate was then collected by centrifugation and analysed by SDS-PAGE.

### Enzyme-linked immunosorbent assay (ELISA)

HSP90 in GC tissues and normal tissues were analyzed by Human HSP90 ELISA Kit (Proteintech, KE00054) according to the manufacturer’s protocol. The absorbance at 450 nm and 630 nm were immediately measured by automatic microplate reader after adding Stop solution.

### Dual luciferase reporter assay

HEK-293 T cells were seeded in 24-well plate at a density of 6 × 10^4^ cells per well for 24 h before transfection. The cells were co-transfected with a mixture of luciferase reporter vectors (pmirGLO) containing circSHKBP1-miR-582/665 binding sequences or mutant sequences and miRNA mimics (20 nM) to examine the miRNA binding ability. After 24 h, the luciferase activity was measured using a dual luciferase reporter assay system (Promega, Madison, WI, USA) according to the manufacturer’s protocol.

### RNA pulldown and mass spectrometry

CircRNA pulldown was performed using Magnetic RNA-protein Pull-down Kit (Thermo), according to the manufacturer’s protocol. 5′Biotin-labeled oligonucleotide probe targeting junction site of circSHKBP1 was synthesized (KeyGEN, Jiangsu, China): 5′-CCTCCTGGACTGGTCTTGGG-3′. A total of 10^7^ HGC27 cells were transfected with circSHKBP1-overexpressing vector or control vector. 48 h later, total RNA from the two groups were extracted and incubated with 100 nmol probe respectively at 70 °C for 5 min. Then RNA was slowly cooled down to room temperature and 50 μl Streptavidin Magnetic Beads was added and incubated at room temperature for 30 min with agitation. Unbound RNA was washed away by 20 mM Tris, and 100 μl 1× RNA-protein binding buffer with 100 μg total protein was added to the tube containing Streptavidin Magnetic Beads. After incubation at 4 °C for 1.5 h with rotation, Streptavidin Magnetic Beads were washed with washing buffer for 3 times, and then incubated with 50 μl Elution buffer at 37 °C for 15 min with agitation. Supernatant was collected for silver staining and mass spectrum by KeyGEN.

### RNA-protein immunoprecipitation (RIP)

The MagnaRIP RNA-Binding Protein Immunoprecipitation Kit (Merck Millipore) was used according to the manufacturer’s instructions. The cell lysates were incubated with beads coated with 5 μg of antibody against Argonaute-2 (AGO2) (Abcam, MA, USA), anti-HSP90 (Proteintech) or anti-HUR (Proteintech) and control IgG with rotation at 4 °C overnight. Next, total RNA was retrieved for the detection of circRNAs and miRNA expression by qRT-PCR.

### RNA in situ hybridization (ISH)

BaseScope™ Reagent Kit v2-RED (Advanced Cell Diagnostics, CA, USA) was used for ISH following the user manual. Briefly, sections were cut at 4 μm thickness onto Superfrost plus slides (Thermo Scientific, New Hampshire, USA) and allowed to dry overnight at room temperature (RT). Sections were then baked at 60 °C for one h before being deparaffinized in xylene (2 × 5 min) and ethanol (2 × 2 min), then dried by baking at 60 °C for 2 min. Pretreat 1 (hydrogen peroxide) was applied for 10 min at RT, Pretreat 2 (target retrieval) for 15 min at 100 °C and Pretreat 3 (protease IV) for 30 min (tissue sections) at 40 °C, with two rinses in distilled water between pretreatments. BaseScope probe (BA-Hs-SHBP1-circRNA, 1zz targeting 291–12 of NC_000019.10:40583398–40,583,717) was then applied for 2 h at 40 °C in a HybEZ oven before incubation with reagents AMP1 (30 min at 40 °C), AMP2 (30 min at 40 °C), AMP3 (15 min at 40 °C), AMP4 (30 min at 40 °C), AMP5 (30 min at 40 °C), AMP6 (15 min at 40 °C), AMP7 (30 min at RT) and AMP8 (15 min at RT). Slides were rinsed with wash buffer (2 × 2 min) between AMP incubations. Finally, slides were incubated with Fast Red for 10 min at RT in the dark. Then, slides were counterstained with hematoxylin before drying for 15 min at 60 °C.

### Biotin-labeled miRNA capture

The miRNA pulldown assay was performed using biotinylated miR-582/665 mimic or control RNA (RiboBio) transfected into HEK-293 T cells at a final concentration of 20 nM for 24 h. The biotin-coupled RNA complex was pulled down by incubating the cell lysates with Streptavidin Magnetic Beads (Thermo). The abundance of circSHKBP1 in bound fractions was evaluated by qRT–PCR.

### Exosome isolation and identification

Exosomes from cells were collected from 20 ml of culture media (1× 10^7^ cells). The media were collected on ice, centrifuged at 800×g for 10 min to sediment the cells, and then centrifuged at 12,000×g for 30 min to remove the cellular debris. Exosomes were separated from the supernatant by centrifugation at 100,000×g for 2 h in a SW32 rotor (Beckman Coulter). The exosome pellet was washed once in a large volume of PBS and resuspended in 100 μl of PBS.

Human serum exosomes were obtained with ExoQuick Exosome Precipitation Solution (SBI, CA, USA) following the user manual. Briefly, serum was collected and centrifuged at 3000×g for 15 min. Then add the 63 μl ExoQuick Exosome Precipitation Solution to 250 μl supernatant and refrigerate the mixture 30 min serum at 4 °C. After centrifugation at 1500×g for 30 min, resuspend exosome pellet in 100 μl using sterile 1× PBS.

Exosomes were then identified by Transmission Electron Microscope (TEM) (Philips TECNAI 20, Netherland) for particle size and form. Exosome protein markers were identified by western blot assay. The total amount of exosomes was detected by nanoparticle tracking analysis (NTA).

### Tube formation assay

96-well plates were coated with 50 μl of Matrigel in each well and incubated to polymerize at 37 °C for 1 h. HUVECs cells were harvested and suspended in fresh complete medium at the density of 4 × 10^5^ /ml. 100 μl cell suspension was seeded on the surface of polymerized Matrigel in 96-well plates and incubated at 37 °C for 4 h. Three or more random pictures of each well were taken with a digital camera system (Olympus, Tokyo, Japan), and total tubule length and number of branches were analyzed by ImageJ software.

### Animal studies

All animal experiments were performed in accordance with a protocol approved by the Institutional Animal Care and Use Committee of Nanjing Medical University (IACUC-1902006).

BGC823 cells that stably expressed or silenced circSHKBP1 and control cells were harvested and suspended in PBS on ice. Eighteen mice (male BALB/c nu/nu, 5 weeks old) were divided randomly into 3 groups, and each mouse was injected subcutaneously in the right thigh root with cells (4 × 10^6^ /150 μl) that inhibited expression of circSHKBP1 (LV3-NC, LV3-sh1 and LV3-sh2). The mice were monitored for body weight and tumor volume (volume = length ×width^2^/2) every 3 days for 24 days after injection. Twenty mice (male BALB/c nu/nu, 5 weeks old) were divided randomly into 2 groups, and one group was injected with cells (4 × 10^6^ /150 μl) that overexpressed circSHKBP1 (LV5-circSHKBP1) and another group was injected with control cells (LV5-NC). The group of 10 mice was then divided into 2 random subgroups according to the volume of xenograft tumors upon the occurrence of solid tumors, and 100 μl PBS or 2 μg/g bevacizumab (Avastin®, Roche, Swiss) were then administrated by intraperitoneal injection. The mice were monitored for 21 days. At the end of experiments, the mice were sacrificed, and the tumors were dissected and weighed. Tumors were used for H&E staining, Western blot, IHC and IF assays. Blood samples were collected for Western blot, blood biochemistry and exosome analysis.

For the in vivo tumor metastasis studies, 56 mice (male BALB/c nu/nu, 5 weeks old) were randomly divided into 7 groups (LV3-NC, LV3-sh1, LV3-sh2, LV5-NC, LV5-circSHKBP1, LV5-NC + BEV, LV5-circSHKBP1 + BEV) to receive tail vein injection (1.5 × 10^6^ cells in 150 μl of PBS). Bevacizumab was administrated instantly after injection. The mice were monitored for body weight every week. Three weeks later, lung metastases were examined by bioluminescence imaging every week for 4 weeks. D-luciferin sodium salt stock solution was prepared in PBS at 15 mg/ml. To produce bioluminescence, the mice received an intraperitoneal injection of luciferin stock solution (150 mg/kg). All mice were immediately anaesthetized with 2% isoflurane and were imaged after 10 min. The images were captured using an IVIS Spectrum Xenogen Imaging System (Caliper Life Sciences). After 7 weeks, all mice were killed and their lungs were resected for H&E staining, Western blot and IHC assays.

### Statistical analysis

The results were reported as the means ± SD of at least three independent experiments. Each exact n value is indicated in the corresponding figure legend. Unless otherwise stated, Student’s t-test and one-way ANOVA were used to determine the statistical significance for comparisons of 2 or more groups. All statistical analyses were performed using SPSS software, version 20.0 (SPSS Inc., USA) and GraphPad Prism, version 7.00 (GraphPad Software, USA). The correlation of the expressions of circSHKBP1 and miR-582 was established by Pearson correlation coefficient and linear regression model. For survival analysis, the median expression level (50th percentile) of circSHKBP1 was used as the cutoff value. Kaplan Meier survival curves were compared using the log-rank test with GraphPad Prism software. *P*-values < 0.05 were considered statistically significant.

## Results

### Identification of circRNAs via RNA-seq in GC

**Fig. 1:**
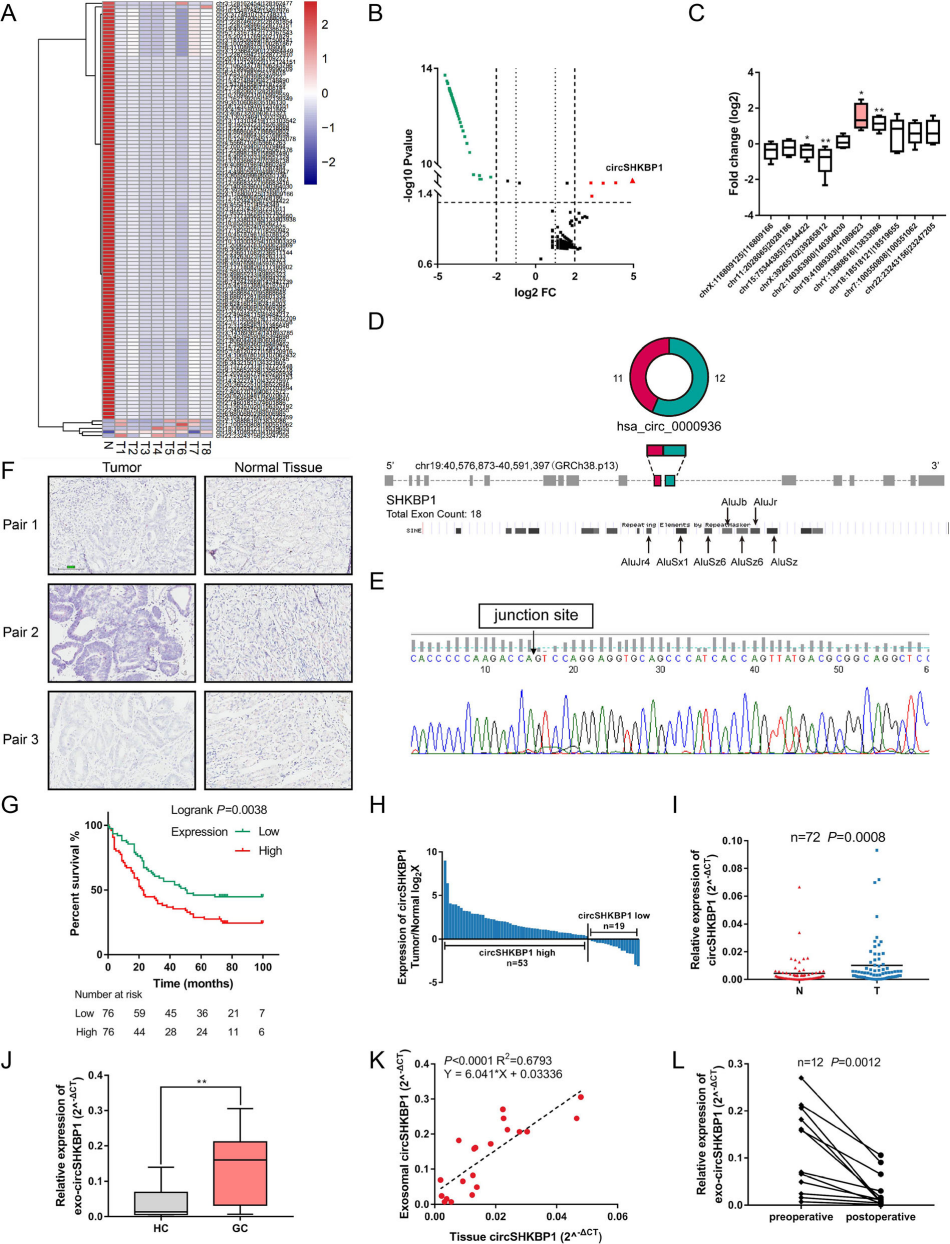
Identification of circSHKBP1 as a biomarker for GC. **a** Cluster heat map showing the differentially expressed circRNAs in paired human GC tissues and normal tissues (*n* = 8). The circRNAs were classified according to the Pearson correlation test. **b** Volcano plot showing circRNAs that changed significantly between GC tissues and matched normal tissues. **c** Fold change of 5 most upregulated and downregulated circRNAs in 10 paired GC tumors and normal tissues detected by qRT-PCR. **d** Structure of circSHKBP1 and ALU elements in the flanking sequence. **e** Back-splicing sequence and junction site of circSHKBP1. **f** Representatives showing the expression of circSHKBP1 (red) in GC tumors and matched normal tissues detected by ISH. **g** Survival analysis of GC patients according to circSHKBP1 expression detected by ISH (*n* = 152, median survival (months): low expression vs. high expression = 50 vs. 22). The median expression level of circSHKBP1 was used as the cutoff value. Log-rank tests were used to determine statistical significance. **h** and **i** Expression of circSHKBP1 in GC tumors was 2.31 times higher than that in normal tissues (*n* = 72, cases: upregulated vs. downregulated = 53 vs. 19, *P* = 0.0008). **j** Level of circSHKBP1 in serum exosomes of GC patients (*n* = 20) and healthy controls (HC) (*n* = 20), as determined by qRT-PCR. **k**. Correlation of circSHKBP1 expression between GC tumors and serum exosomes (Y = 6.041*X + 0.03336, R^2^ = 0.6793, *P* < 0.0001). **l** Levels of exosomal circSHKBP1 before and after gastrectomy (*n* = 12). Quantitative data from three independent experiments are shown as the mean ± SD (error bars). *P < 0.05, **P < 0.01, ***P < 0.001 (Student’s t-test)

The RNA-seq data were first mapped using CIRI [25] to the human reference genome (GRCh37/hg19) obtained from the UCSC genome database (http://genome.ucsc.edu/). The normalized intensity (log2-transformed) of each sample was used as an absolute measure of circRNA abundance. Through RNA-seq of 8 GC tissues and one mixed sample of 8 matched adjacent normal tissues, we identified a total of 1445 distinct circRNA candidates with at least 2 unique back-splicing junction reads. Among these circRNAs, 557 were intergenic, 378 were antisense, 81 were sense overlapping, 20 were exonic and 409 were intronic. A total of 119 circRNAs were differentially expressed (FC (fold change) ≥4 and *P* < 0.05) between cancerous tissues and normal tissues, among which 5 circRNAs were upregulated and 114 were downregulated in GC compared to normal tissues (Fig. 1a and b).

Then, we further analyzed the expression of top 5 upregulated and downregulated circRNAs in 10 paired GC tumors and normal tissues by qRT-PCR and found that circSHKBP1 was the most differentially expressed circRNA in GC (Fig. 1c).

### Characterization of circSHKBP1 and its expression in GC

CircSHKBP1 (hsa_circ_0000936, chr19:40,576,873-40,591,397 (GRCh38.p13)) was derived from the protein-coding locus SHKBP1, which is widely expressed in human tissues. CircSHKBP1 was first identified in 2013 [26, 27] and was found to be highly abundant in many endothelial cells [28]. CircSHKBP1 is generated by back splicing of the 11th and 12th exons of the SHKBP1 gene with several Alu elements in the introns on both sides (Fig. 1d and S1A). We designed convergent primers across the junction site and confirmed the reverse transcription product by Sanger sequencing (Fig. 1e). Resistance to digestion by RNase R and a lower degradation rate compared with SHKBP1 mRNA verified that circSHKBP1 exists as a circular form and is highly stable with a half-life period of over 24 h (Fig. S1D and S1E).

Then, we analyzed the expression of circSHKBP1 in 72 paired GC tissues and normal tissues. qRT-PCR analysis showed that circSHKBP1 was upregulated in 53 GC tissues compared with the matched normal tissues (Fig. 1h), and the expression of circSHKBP1 was 2.31-fold higher in GC tissues on average than in normal tissues (*P* < 0.05) (Fig. 1i). Moreover, we performed an ISH assay in a tissue microarray with 152 pairs of cancerous and normal tissues from GC patients. The results showed that the abundance of circSHKBP1 in GC tissues was much higher than that in matched normal tissues (Fig. 1f), and the expression of circSHKBP1 was correlated with advanced TNM stage, vascular invasion and poor prognosis (Fig. 1g and Table 1).

To determine whether circSHKBP1 can be detected in serum exosomes, we collected blood samples from 20 GC patients and 20 healthy controls. The exosomes derived from serum were identified by TEM and western blot assay of protein biomarkers (Fig. S1F). As expected, circSHKBP1 derived from serum exosomes was more abundant in GC patients than in healthy controls (Fig. 1j). Moreover, the levels of circSHKBP1 in serum exosomes were consistent with those in tumors of GC patients and were approximately 6 times higher than those in tumors (Fig. 1k), making it possible to detect the expression of circSHKBP1 from blood samples. We also investigated the expression of exosomal circSHKBP1 in serum before and after gastrectomy (R0 resection) and found that circSHKBP1 sharply decreased after the removal of tumors (*n* = 12), which indicated GC tissues were the origin of exosomal circSHKBP1 (Fig. 1l).

These results suggest that circSHKBP1 is an upregulated circRNA derived from GC tissues and can be effectively delivered by exosomes into the circulation. Moreover, a high expression level of circSHKBP1 is associated with advanced TNM stage and poor prognosis of GC, making it a potential promising RNA biomarker of GC.

### CircSHKBP1 promotes GC cell proliferation, migration and invasion in vitro

**Fig. 2:**
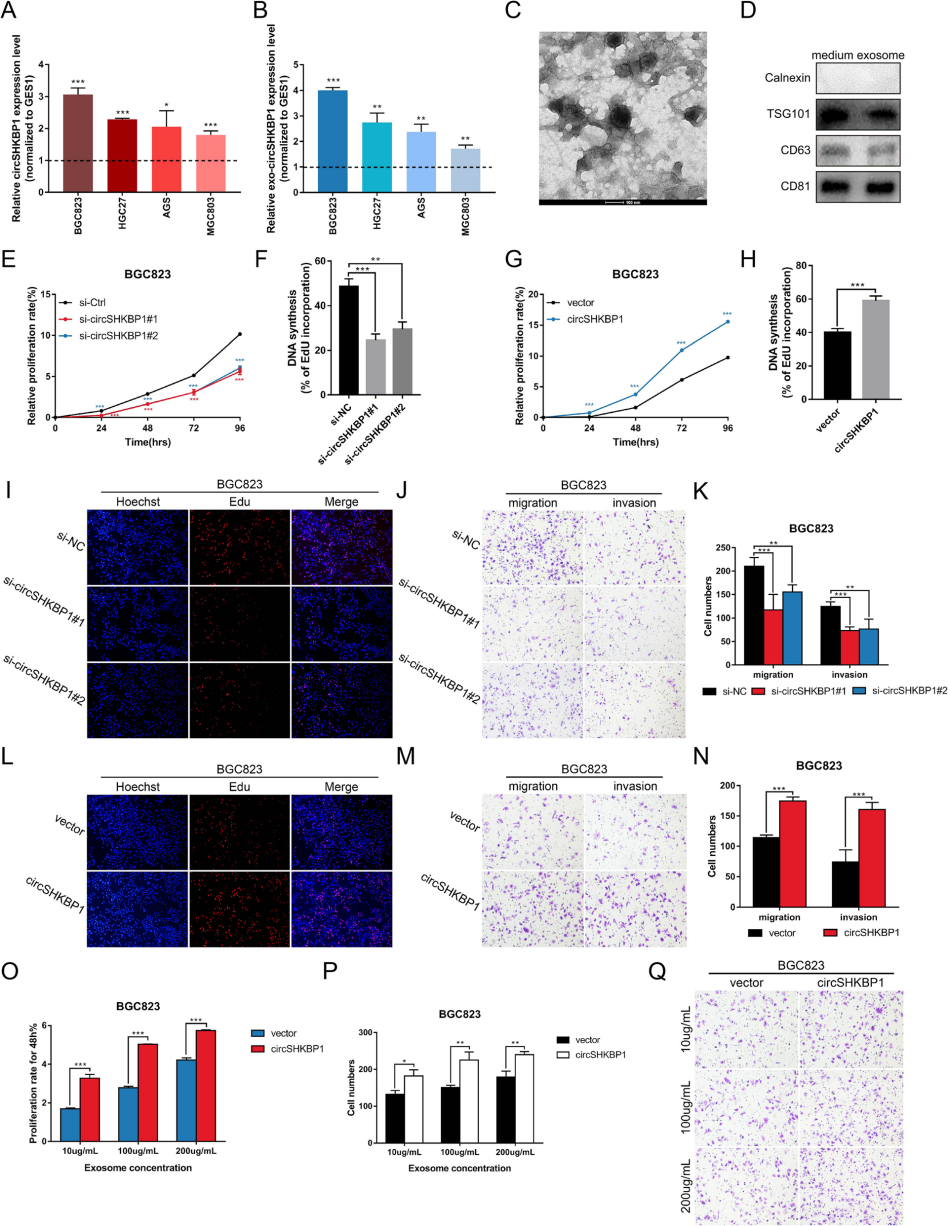
CircSHKBP1 promotes GC cell proliferation, migration and invasion in vitro. **a** and **b** Level of circSHKBP1 in BGC823, HGC27, AGS, MGC803 and GES1 cells and their medium-derived exosomes, as determined by qRT-PCR (normalized to GES1 levels). **c** and **d** Identification of medium exosomes of BGC823 cells by TEM and western blot. **e** Assessment of the proliferation of BGC823 cells transfected with control or circSHKBP1 siRNAs by CCK8 assay. **f** and **i** Assessment of the proliferation of BGC823 cells transfected with control or circSHKBP1 siRNAs by EdU assay. **g** Assessment of the proliferation of BGC823 cells transfected with control vector or circSHKBP1 plasmid by CCK8 assay. **h** and **l** Assessment of the proliferation of BGC823 cells transfected with control vector or circSHKBP1 plasmid by EdU assay. **j** and **k** Assessment of the migration and invasion of BGC823 cells transfected with control or circSHKBP1 siRNAs by Transwell assay. **m** and **n** Assessment of the migration and invasion of BGC823 cells transfected with control vector or circSHKBP1 plasmid by Transwell assay. **o** Assessment of the proliferation of BGC823 cells cocultured with circSHKBP1-overexpressing exosomes or normal exosomes for 48 h at different concentrations by CCK8 assay. **p** and **q** Assessment of the migration of BGC823 cells cocultured with circSHKBP1-overexpressing exosomes or normal exosomes by Transwell assay. Quantitative data from three independent experiments are shown as the mean ± SD (error bars). *P < 0.05, **P < 0.01, ***P < 0.001 (Student’s t-test)

To investigate whether circSHKBP1 affects the biological processes of GC cells, we first analyzed the expression of circSHKBP1 in 4 human GC cell lines (BGC823, HGC27, AGS and MGC803) and the normal gastric epithelial cell line GES1. Compared to the level in GES1 cells, circSHKBP1 was overexpressed in all 4 GC cell lines, especially BGC823 and HGC27(FC = 3.07 and 2.29, *P* < 0.001) (Fig. 2a). Exosomes derived from BGC823 cell culture medium were identified, and similarly, exosomal circSHKBP1 was also overexpressed in GC cell lines compared with GES1 cells (BGC823: FC = 4.00, *P* < 0.001; HGC27: FC = 2.74, *P* < 0.01) (Fig. 2b-d). However, the expression of SHKBP1 linear mRNA showed no significant difference between the cell lines (Fig. S2A). Therefore, we used BGC823 and HGC27 cells in the following analyses.

We designed 2 siRNAs targeting the back-spliced sequence of circSHKBP1, so they would not affect SHKBP1 linear mRNA expression. We tested the efficiency of si-circSHKBP1#1 and si-circSHKBP1#2 and found that they significantly inhibited the expression of circSHKBP1 but not that of SHKBP1 mRNA (Fig. S2B and S2C). CCK8 and EdU assays revealed that silencing circSHKBP1 remarkably inhibited GC cell proliferation (Fig. 2e, f, i, S2F, S2G and S2J). Transwell assays showed that silencing circSHKBP1 suppressed GC cell migration and invasion abilities (Fig. 2j, k, S2K and S2L).

We constructed a circSHKBP1 overexpression plasmid using the pcDNA3.1-CMV-circRNA vector (Fig. S2D and S2E). CCK8, EdU and Transwell assays revealed that overexpression of circSHKBP1 increased GC cell proliferation, migration and invasion (Fig. 2g, h, l-n, S2H, S2I and S2M-O).

qRT-PCR analysis revealed that the expression of exo-circSHKBP1 changed consistently with cellular circSHKBP1 after transfection (Fig. S2P and S2Q). The total amount of exosomes was detected by NTA and the result showed that the release of exosomes had a slight increase (concentration (*10^9^ particles/mL): vector vs. circSHKBP1 = (20.5 ± 2.45) vs. (14.5 ± 0.90), *P* = 0.082) after transfection of circSHKBP1 plasmid (Fig. S2R). However, in consideration of the significant overexpression of exosomal circSHKBP1, we attributed the increase of exosomal circSHKBP1 to more circSHKBP1 being loaded into exosomes instead of more exosomes being released. We extracted exosomes from the culture medium of circSHKBP1 plasmid-transfected BGC823 and HGC27 cells and cocultured them with untreated GC cells at different concentrations. As expected, overexpression of exosomal circSHKBP1 also affected GC cell proliferation, migration and invasion, promoting malignant cell phenotypes (Fig. 2o-q).

These results indicate that circSHKBP1 promotes GC cell growth and metastasis, while silencing circSHKBP1 expression inhibits GC cell development. More importantly, with the ectopic expression of circSHKBP1 in GC cells, more circSHKBP1 is loaded into exosomes, thus interfering with the biological functions of adjacent or distant GC cells.

### CircSHKBP1 serves as a sponge of miR-582-3p

**Fig. 3:**
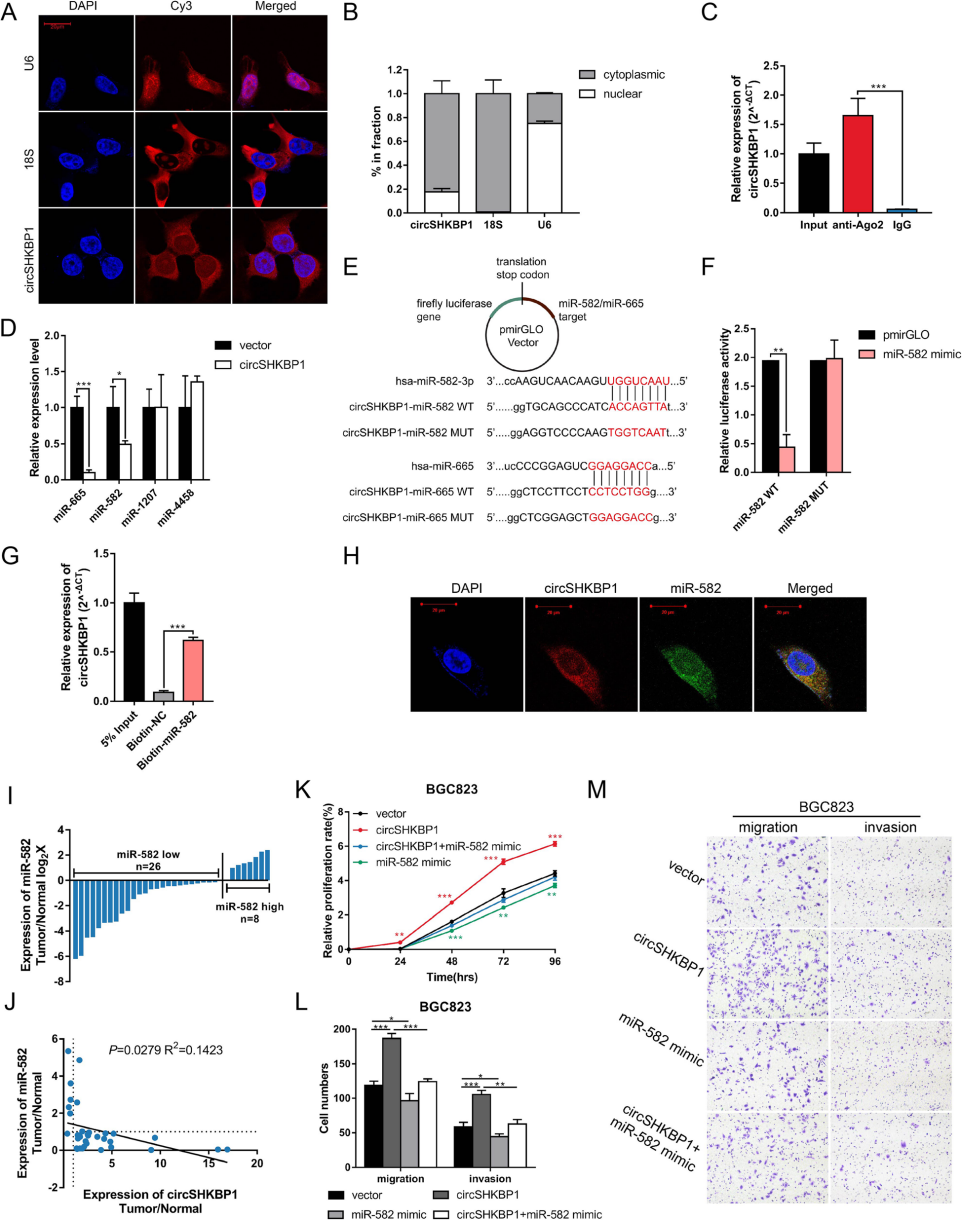
CircSHKBP1 serves as a sponge of miR-582-3p. **a** Localization of circSHKBP1 in HGC27 cells detected by FISH. U6 and 18S rRNA were used as positive controls for the nuclear and cytoplasmic fractions, respectively. **b** Abundance of circSHKBP1 in either the cytoplasm or nucleus of HGC27 cells detected by qRT-PCR. **c** Level of circSHKBP1 detected by qRT-PCR after RIP for Ago2 in BGC823 cells. **d** Levels of miR-582, miR-665, miR-1207 and miR-4458 in BGC823 cells transfected with control vector or circSHKBP1 plasmid detected by qRT-PCR. **e** Structure of the pmirGLO vector and schematic graph of potential binding sites between circSHKBP1 and miR-582 or miR-665. **f** Dual luciferase reporter assay used to detect the relative luciferase activity (firefly/renilla) in HEK-293 T cells cotransfected with miR-582 mimics and pmirGLO-circSHKBP1-miR-582 WT/MUT. **g** Level of circSHKBP1 pulled down by biotin-labeled miR-582 or control probe. (H). Colocalization of circSHKBP1 and miR-582-3p in HGC27 cells detected by FISH. **i** Expression of miR-582 in paired GC tumors and normal tissues (*n* = 34). **j** Correlation of circSHKBP1 and miR-582 expression in GC tissues (R^2^ = 0.1423, *P* = 0.0279). **k** Assessment of the proliferation of BGC823 cells transfected with control vector, circSHKBP1 plasmid, or miR-582 mimic or cotransfected with miR-582 mimic and circSHKBP1 plasmid by CCK8 assay. **l** and **m** Assessment of the migration and invasion of BGC823 cells transfected with control vector, circSHKBP1 plasmid, or miR-582 mimic or cotransfected with miR-582 mimic and circSHKBP1 plasmid by Transwell assay. Quantitative data from three independent experiments are shown as the mean ± SD (error bars). *P < 0.05, **P < 0.01, ***P < 0.001 (Student’s t-test)

To investigate the mechanism by which circSHKBP1 functions in GC cells, we first confirmed its intracellular localization. FISH assay and qRT-PCR analysis of nuclear and cytoplasmic RNA demonstrated that circSHKBP1 preferentially localized within the cytoplasm of GC cells (Fig. 3a and b). Given that circRNAs have been widely explored as miRNA sponges and circSHKBP1 is abundant in the cytoplasm, we next investigated the miRNA binding ability of circSHKBP1. We conducted RIP for AGO2 in BGC823 cells and observed that endogenous circSHKBP1 pulled down in BGC823 cells was specifically enriched by qRT–PCR analysis (Fig. 3c), indicating that circSHKBP1 acted as a miRNA sponge. Four potential miRNAs (miR-582-3p, miR-665, miR-1207 and miR-4458) were predicted to bind to circSHKBP1 by CircInteractome (circinteractome.nia.nih.gov). We detected the expression of these miRNAs in circSHKBP1-overexpressing GC cells and found that miR-582-3p and miR-665 were downregulated (Fig. 3d). To verify the direct interaction between miR-582-3p/miR-665 and circSHKBP1, we constructed a circSHKBP1 fragment containing the predicted binding site (wild type and mutant) of the identified miRNA and inserted it downstream of the dual luciferase reporter gene (Fig. 3e). The results demonstrated that the miR-582-3p mimic caused downregulation of the relative luciferase activity of the circSHKBP1-miR-582-3p groups compared with the miR-NC mimic, while the luciferase activity of the circSHKBP1-miR-582-3p mutant group did not change (Fig. 3f). However, the miR-665 mimic did not affect the luciferase activity of circSHKBP1-miR-665 (Fig. S3A). A biotin-labeled RNA pulldown assay was performed, which confirmed the absorption of circSHKBP1 and miR-582-3p but not miR-665 (Fig. 3g and S3B). Moreover, FISH assay confirmed that circSHKBP1 and miR-582-3p colocalized in cytoplasm (Fig. 3h), indicating the binding of them. qRT-PCR showed that the level of miR-582-3p was lower in GC tumors than in paired normal tissues, and the expression of miR-582-3p and circSHKBP1 was negatively correlated (Fig. 3i and j). These results suggest that circSHKBP1 acts as a sponge of miR-582-3p and decreases the expression of miR-582-3p.

To confirm the biological functions of miR-582-3p and whether circSHKBP1 affects the function of miR-582-3p, we transfected miR-582-3p mimic in GC cells alone or together with the circSHKBP1 plasmid (Fig. S3C and S3D). CCK8 and Transwell assays showed that the miR-582-3p mimic suppressed GC cell proliferation, migration and invasion, and circSHKBP1 overexpression abrogated this inhibition of GC cell development (Fig. 3k-m and S3E-G).

### CircSHKBP1 promotes VEGF translation by upregulating HUR

**Fig. 4:**
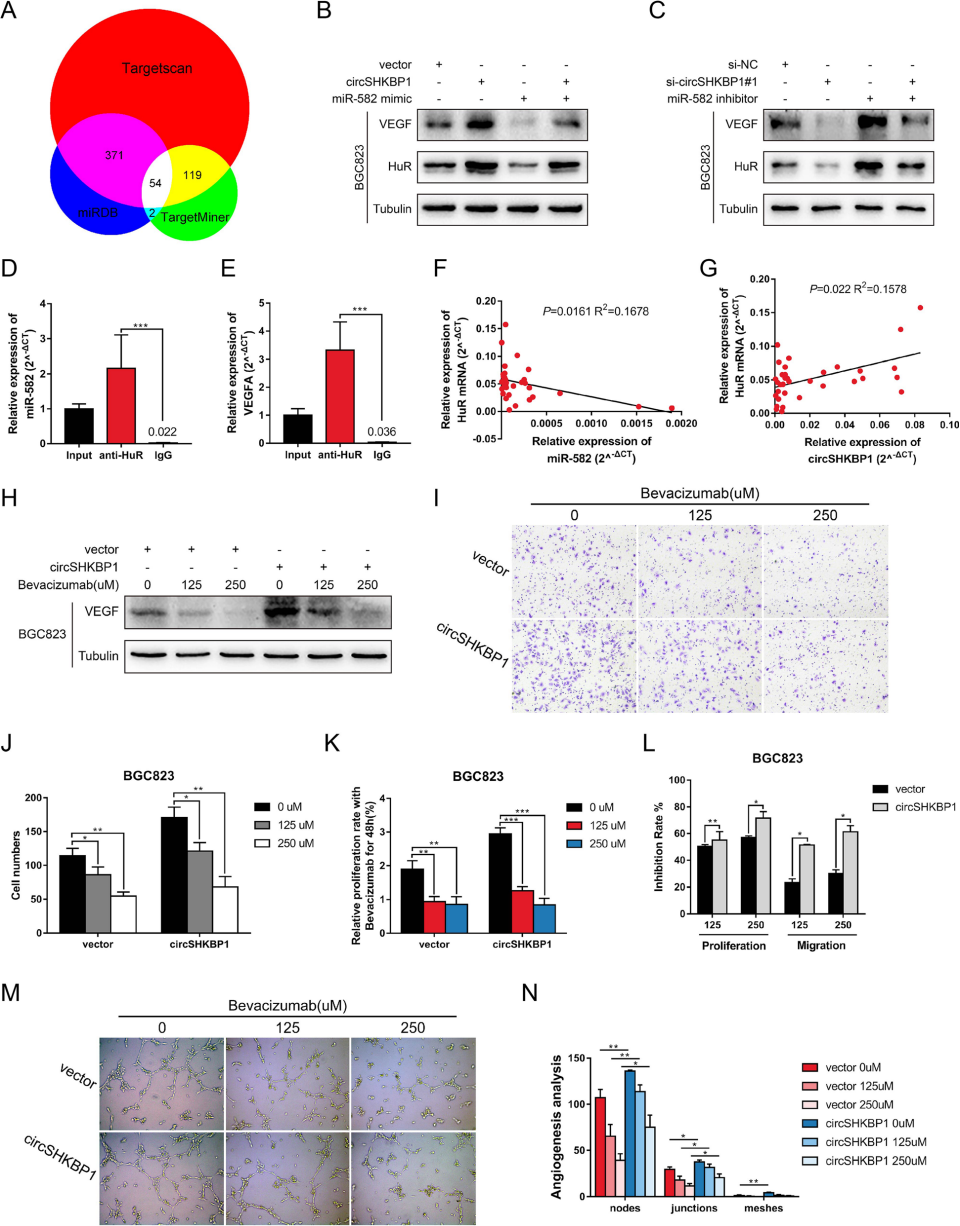
CircSHKBP1 promotes VEGF translation by upregulating HUR. **a** Venn diagram showing targets of miR-582 predicted from TargetScan, TargetMiner and miRDB. **b** Western blot showing the expression levels of VEGF and HUR in BGC823 cells transfected with control vector, circSHKBP1 plasmid, or miR-582 mimic or cotransfected with miR-582 mimic and circSHKBP1 plasmid (tubulin as an internal control). **c** Western blot showing the expression levels of VEGF and HUR in BGC823 cells transfected with control, circSHKBP1 siRNAs, or miR-582 inhibitor or cotransfected with miR-582 inhibitor and circSHKBP1 siRNAs (tubulin as an internal control). **d** and **e** Levels of miR-582 and VEGF mRNA detected by qRT-PCR after RIP for HUR in BGC823 cells. **f** Correlation of HUR and miR-582 expression in GC tissues (R^2^ = 0.1678, *P* = 0.0161). **g** Correlation of HUR and circSHKBP1 expression in GC tissues (R^2^ = 0.1578, *P* = 0.022). **h** Western blot showing the expression level of VEGF in BGC823 cells transfected with control vector or circSHKBP1 plasmid treated with bevacizumab at different concentrations. **i** and **j** Assessment of the migration of BGC823 cells transfected with control vector or circSHKBP1 plasmid and treated with bevacizumab at different concentrations, as determined by Transwell assay. **k** Assessment of the proliferation of BGC823 cells transfected with control vector or circSHKBP1 plasmid and treated with bevacizumab at different concentrations, as determined by CCK8 assay. **l** Inhibition rate of bevacizumab of the proliferation and migration of BGC823 cells transfected with control vector or circSHKBP1 plasmid. **m** and **n** Tube formation assay of BGC823 cells transfected with control vector or circSHKBP1 plasmid and treated with bevacizumab at different concentrations. Angiogenesis analysis of tube nodes, junctions and meshes as measured by ImageJ. Quantitative data from three independent experiments are shown as the mean ± SD (error bars). *P < 0.05, **P < 0.01, ***P < 0.001 (Student’s t-test)

By analyzing the clinical data in Table 1, we found that the vascular invasion rate in high circSHKBP1 group (39.5%, 30 out of 76) was much higher than that in low circSHKBP1 group (23.7%, 18 out of 76). With the bioinformatics analysis websites (TargetScan, TargetMiner and miRDB), we found 54 miR-582-3p target protein candidates (Fig. 4a), of which HUR and EIF2S1 have been reported to be part of the VEGF signaling pathway. VEGF is one of the most important factors promoting endothelial cell proliferation, so we conducted in-depth research on the VEGF pathway. According to TCGA database, HUR and EIF2S1 are overexpressed in GC (Fig. S4A and S4B). Survival analysis for GC (data source: KMplot.com) [29] showed that high expression of HUR led to shorter OS (median survival (months): low expression cohort vs. high expression cohort = 35.2 vs. 25.9, *P* < 0.0001) (Fig. S4C), while survival analysis for EIF2S1 showed no significant difference between the low expression and high expression cohorts (*P* = 0.41) (Fig. S4D). Overexpression of circSHKBP1 increased the levels of HUR and VEGF (Fig. 4b and S4F), as demonstrated by western blot assay, while it had no effect on the level of EIF2S1 (Fig. S4E). Moreover, the increased HUR and VEGF protein levels were decreased by miR-582-3p mimic (Fig. 4b and S4F). Similarly, silencing circSHKBP1 decreased the levels of HUR and VEGF, which were recovered by miR-582-3p inhibitor (Fig. 4c and S4F). HUR is an important element in the VEGF signaling pathway by binding to the AREs of VEGF mRNA, thus stabilizing the mRNA structure and inducing VEGF translation [30, 31].. RIP for HUR showed significant enrichment of VEGF mRNA and miR-582-3p compared with the controls (Fig. 4d and e), further confirming that HUR was the target of miR-582-3p and that HUR directly bound to VEGF mRNA. Data from TCGA database showed that the expression of HUR was negatively related to the expression of miR-582 (Fig. S4G). We also detected the level of HUR mRNA in GC tissues and found that the expression of HUR was negatively related to miR-582-3p while positively related to circSHKBP1 (Fig. 4f and g). We treated circSHKBP1-overexpressing GC cells with different concentrations of bevacizumab, an antibody against VEGF, to determine whether circSHKBP1 accelerates tumor progression via VEGF. Western blot assays showed that the level of VEGF decreased with increasing concentrations of bevacizumab (Fig. 4h and S4H). GC cell proliferation and migration abilities were weakened by treatment with bevacizumab in a concentration-dependent manner (Fig. 4i-k and S4I-K), and the inhibition rate was much higher in circSHKBP1-overexpressing cells than in the control (Fig. 4l and S4L). A tube formation assay further confirmed that circSHKBP1 promoted VEGF secretion and induced angiogenesis, which could be inhibited by bevacizumab (Fig. 4m and n).

### CircSHKBP1 directly interacts with HSP90 and inhibits its degradation

**Fig. 5:**
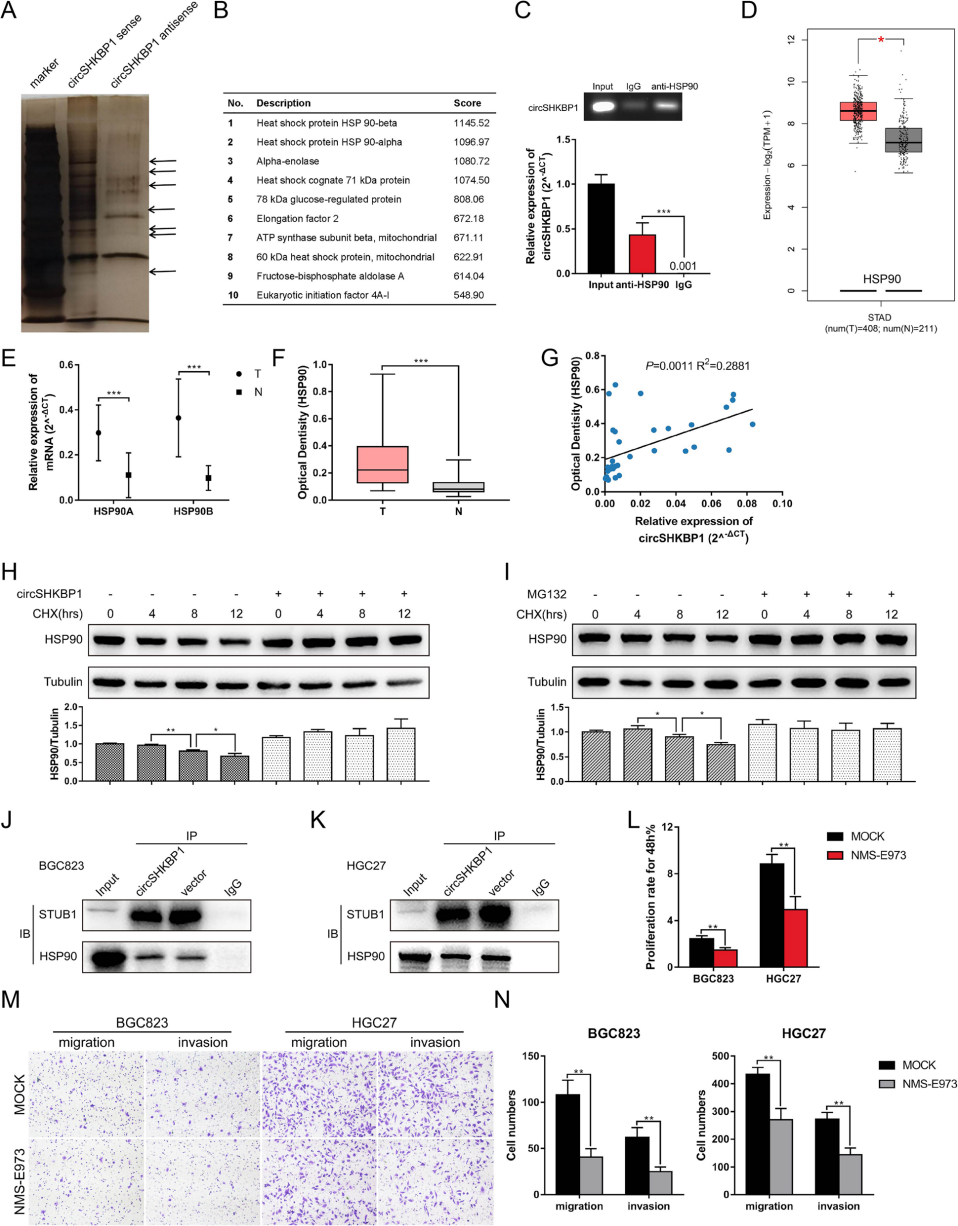
CircSHKBP1 directly interacts with HSP90 and inhibits its degradation. **a**. Silver staining of circSHKBP1 pulldown. Arrows show different bands between the sense and antisense lanes. **b**. List of the top 10 differentially expressed proteins identified by mass spectrometry. **c**. Level of circSHKBP1 detected by qRT-PCR after RIP for HSP90 in BGC823 cells. **d**. Expression of HSP90 in GC tumors (*n* = 408) and normal tissues (*n* = 211) obtained from TCGA database. **e** Levels of HSP90A and HSP90B in paired GC tumors and normal tissues detected by qRT-PCR (*n* = 34). **f** Levels of HSP90 in paired GC tumors and normal tissues detected by ELISA (*n* = 34). **g** Correlation of HSP90 protein and circSHKBP1 expression in GC tissues (R^2^ = 0.2881, *P* = 0.011). **h** Western blot with grey value columns showing the expression of HSP90 in BGC823 cells transfected with control vector or circSHKBP1 plasmid after treatment with CHX (100 μg/ml) for 0 h, 4 h, 8 h, 12 h (tubulin as an internal control). **i** Western blot with grey value columns showing the expression of HSP90 in BGC823 cells treated with or without MG132 after treatment with CHX (100 μg/ml) for 0 h, 4 h, 8 h, 12 h (tubulin as an internal control). **j** and **k** STUB1 immunoprecipitated using an anti-HSP90 antibody. STUB1 was reduced in the immunoprecipitate of BGC823 and HGC27 cells transfected with circSHKBP1. **l** Assessment of the proliferation of BGC823 and HGC27 cells treated with or without HSP90 inhibitor NMS-E973 by CCK8 assay. **m** and **n** Assessment of the migration and invasion of BGC823 and HGC27 cells treated with or without the HSP90 inhibitor NMS-E973 by Transwell assay. Quantitative data from three independent experiments are shown as the mean ± SD (error bars). *P < 0.05, **P < 0.01, ***P < 0.001 (Student’s t-test)

We designed a specific biotin-labeled circSHKBP1 probe to perform an RNA pulldown assay in HGC27 cells. The silver staining results showed enrichment of several bands of proteins in circSHKBP1-overexpressing GC cells compared to the control (Fig. 5a). Protein mass spectrometry analysis was used to identify differentially expressed proteins. In the ranking list of recognized proteins, the top 2 were HSP90β and HSP90α (Fig. 5b), both isoforms of HSP90. The RIP assay revealed that the anti-HSP90 antibody pulled down abundant circSHKBP1 compared to IgG (Fig. 5c), confirming the direct interaction between circSHKBP1 and HSP90. HSP90 was shown to be upregulated in GC by TCGA analysis (Fig. 5d). We also investigated the level of HSP90 mRNA by qRT-PCR assay and the level of HSP90 protein by ELISA in tissues. Results showed that both HSP90 mRNA and protein were upregulated in GC tumors compared to normal tissues (Fig. 5e and f). Given that it was the interaction between circRNA and protein, we analyzed the relationship between the expression of HSP90 protein and circSHKBP1 and found they were positively correlated (Fig. 5g). Western blotting showed that the overexpression of circSHKBP1 slightly increased the total amount of HSP90 (Fig. 5e). However, after suppressing protein synthesis with CHX, the degradation of HSP90 was remarkably suppressed when circSHKBP1 was overexpressed (Fig. 5h). The E3 ubiquitin ligase STUB1 has been demonstrated to ubiquitinate HSP90, thereby targeting it to the proteasome for degradation [32]. Therefore, we administered MG132, an inhibitor of the proteasome, to block the ubiquitination of HSP90. The degradation of HSP90 slowed down under MG132 treatment (Fig. 5i). The hypothesis that circSHKBP1 and STUB1 competitively bound with HSP90 at similar sites was raised to explain how circSHKBP1 protected HSP90 against degradation. An IP assay was performed and the result showed that overexpression of circSHKBP1 decreased the amount of STUB1 binding to HSP90 (Fig. 5j and k), verifying the above hypothesis. Furthermore, NMS-E973, the selective inhibitor of HSP90, impaired the tumor promoting function of circSHKBP1 in vitro (Fig. 5l-n). These results indicate that circSHKBP1 directly bind to HSP90 and suppress the ubiquitination of HSP90 by STUB1, thus accelerating GC development.

### CircSHKBP1 regulates GC growth in vivo

**Fig. 6:**
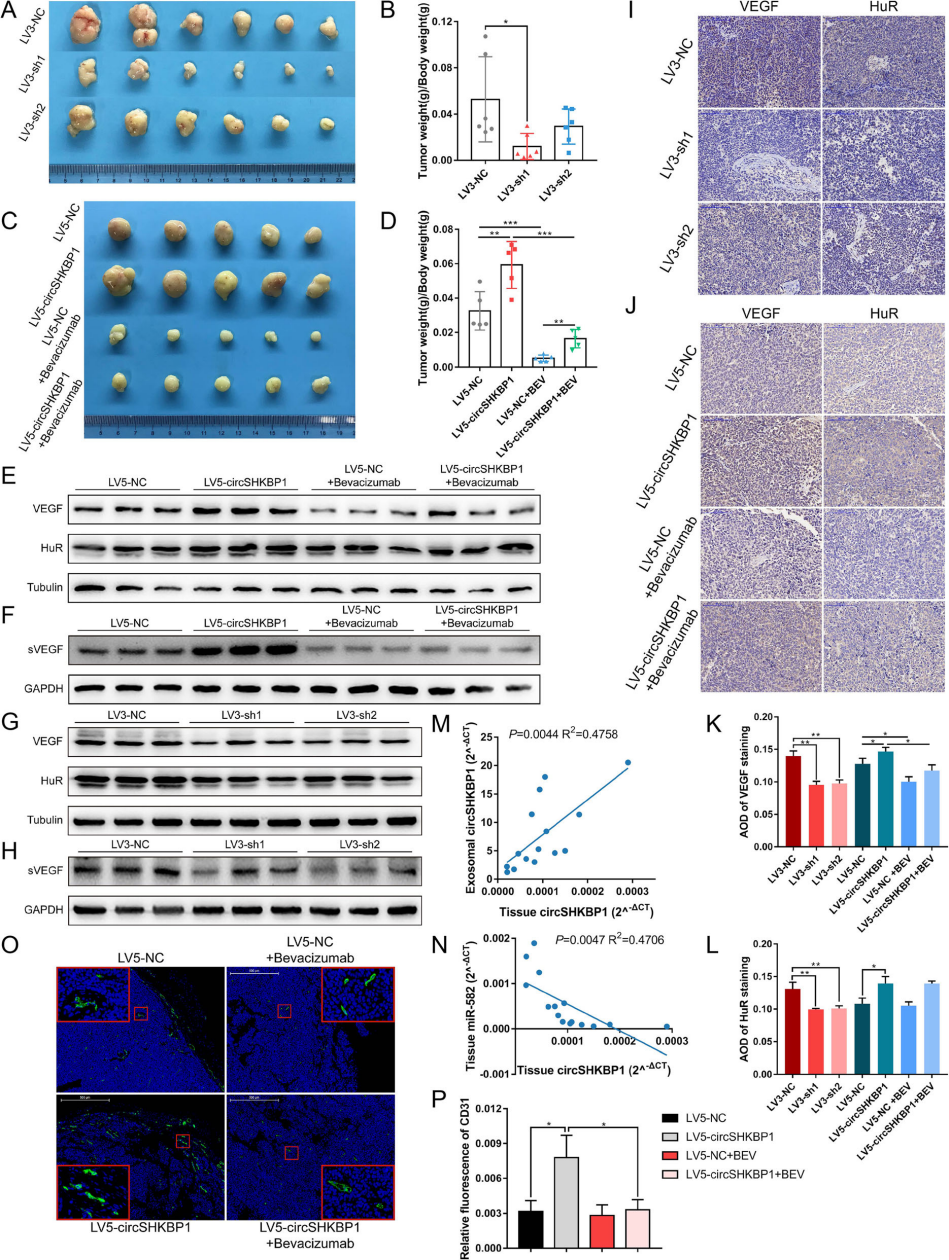
CircSHKBP1 promotes GC growth in vivo. **a** and **b**. Xenograft tumors of nude mice 24 days after injection of BGC823 LV3-NC, LV3-sh1 or LV3-sh2 cells (*n* = 6 per group). Tumor weight and body weight were measured at the end point. **c** and **d**. Xenograft tumors of nude mice 21 days after injection of BGC823 LV5-NC or LV5-circSHKBP1 cells with or without the bevacizumab treatment (*n* = 6 per group). Tumor weight and body weight were measured at the end point. **e** and **f** Western blot showing the expression of HUR and VEGF in tumors (**e**) and serum VEGF (**f**) of LV5-NC and LV5-circSHKBP1 mice with or without bevacizumab treatment (tubulin and GAPDH as internal controls, respectively). **g** and **h** Western blot showing the expression of HUR and VEGF in tumors (**g**) and serum VEGF (**h**) of LV3-NC, LV3-sh1 and LV3-sh2 mice (tubulin and GAPDH as internal controls, respectively). **i** and **j**. IHC of VEGF and HUR in tumors of different groups. **k** and **l**. AOD of VEGF and HUR staining in tumors of different groups. **m**. Correlation of circSHKBP1 expression between xenograft tumors and serum exosomes of mice (R^2^ = 0.4758, *P* = 0.0044). **n**. Correlation of circSHKBP1 and miR-582 expression in xenograft tumors (R^2^ = 0.4706, *P* = 0.0047). **o** and **p**. IF of CD31 in xenograft tumors of LV5-NC and LV5-circSHKBP1 mice with or without bevacizumab treatment. The relative fluorescence of CD31 was measured by ImageJ. Quantitative data from three independent experiments are shown as the mean ± SD (error bars). *P < 0.05, **P < 0.01, ***P < 0.001 (Student’s t-test)

By transducing BGC823 cells with lentiviruses, we constructed the LV3-sh1 and BGC823-LV3-sh2 cell lines with stably silenced circSHKBP1 (Fig. S5A) and the LV5-circSHKBP1 cell line with stably overexpressed circSHKBP1 (Fig. S5B). LV3-sh1, LV3-sh2 and LV3-NC cells were inoculated subcutaneously into the right thighs of nude mice, and these mice were monitored closely for tumor growth for 24 days. The results illustrated that tumors derived from LV3-sh1 cells were significantly smaller than those from LV3-NC cells, both in terms of tumor volume and tumor weight/body weight ratios (Fig. 6a, b, S5C and S5D), suggesting that circSHKBP1 knockdown could limit tumor growth. Additionally, we inoculated LV5-circSHKBP1 and LV5-NC cells subcutaneously into the right thighs of nude mice, and half of the mice in each group were administrated bevacizumab twice per week. After 21 days of monitoring, tumors derived from LV5-circSHKBP1 cells presented a larger size and heavier weight than tumors from LV5-NC cells. Moreover, bevacizumab remarkably inhibited tumor growth (Fig. 6c, d, S5E and S5F). By performing western blot analysis of proteins from mouse serum and tumors, we found that HUR and VEGF levels decreased in the LV3-sh1 and LV3-sh2 groups compared to the LV3-NC group (Fig. 6g and h); HUR and VEGF increased in the LV5-circSHKBP1 group compared to the LV5-NC group, while VEGF was repressed by bevacizumab (Fig. 6e and f). IHC of HUR and VEGF in tumors showed similar results (Fig. 6i-l). Next, we extracted total RNA from serum exosomes and tumors to investigate circSHKBP1 by qRT-PCR. The results demonstrated that exosomal circSHKBP1 expression was linearly dependent on cancer tissue circSHKBP1 expression, and was 61,713-fold more abundant on average (Fig. 6m), confirming that circSHKBP1 could be delivered to distal sites via exosomes and detected in circulation, and its abundance in blood made it a promising biomarker for GC. We also measured the level of miR-582-3p in tumors and found that it was negatively correlated with the level of circSHKBP1 (Fig. 6n). Moreover, IF of CD31 in tumors showed that the florescence area in the LV5-circSHKBP1 group was much more extensive than that in the controls (Fig. 6o and p), indicative of active angiogenesis, and bevacizumab effectively inhibited angiogenesis induced by circSHKBP1. We tested the serum biochemical indexes of mice and found no significant difference between the groups (Additional file 3).

### CircSHKBP1 regulates GC metastasis in vivo

**Fig. 7:**
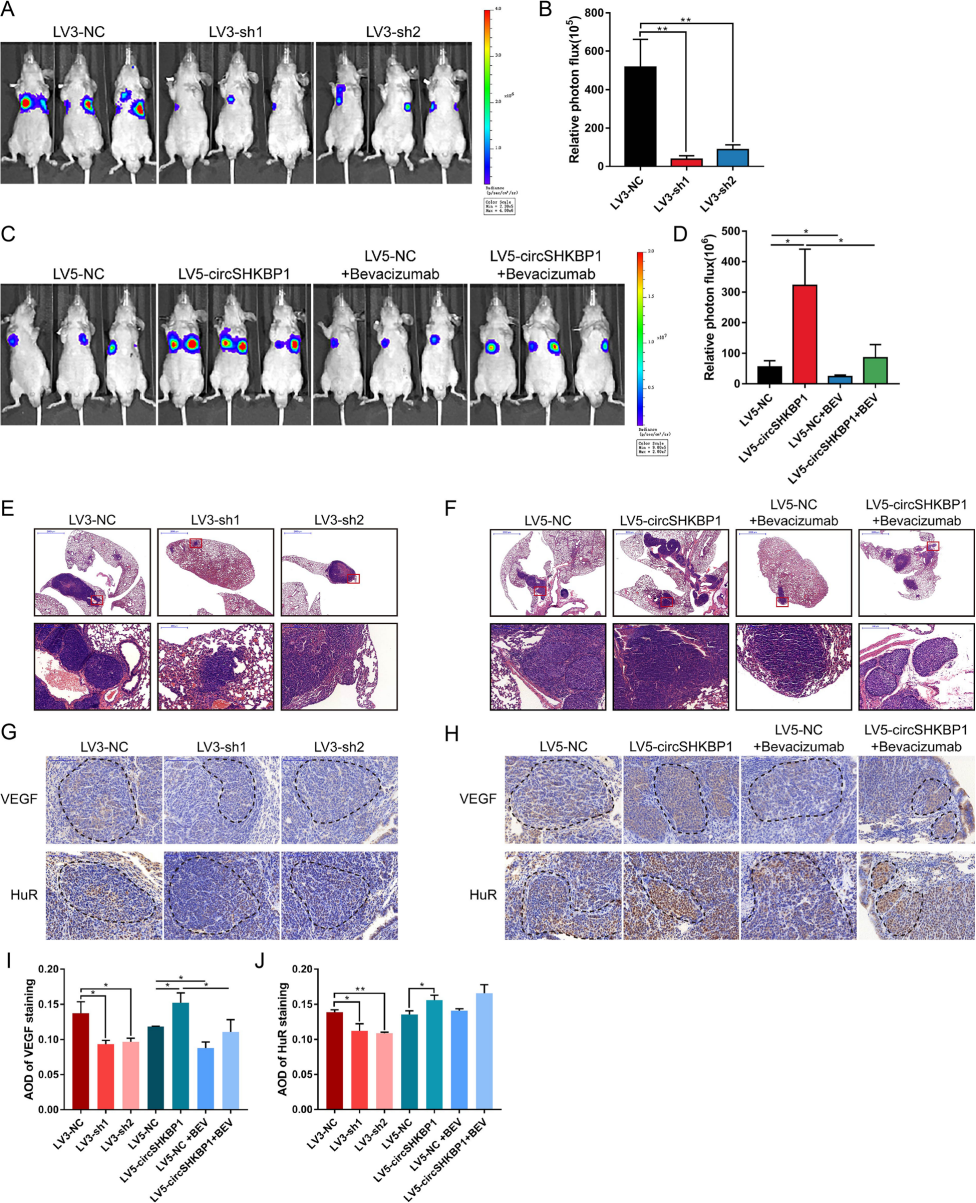
CircSHKBP1 promotes GC metastasis in vivo. **a** and **b**. Bioluminescence imaging (BLI) of mice 7 weeks after tail vein injection of BGC823 LV3-NC, LV3-sh1 or LV3-sh2 cells (*n* = 5 per group). **a** Representative images. **b** Quantification of BLI in the lung region. **c** and **d**. BLI of mice 7 weeks after tail vein injection of BGC823 LV5-NC or LV5-circSHKBP1 cells with or without bevacizumab treatment (*n* = 5 per group). **c** Representative images. **d** Quantification of BLI in the lung region. **e** and **f**. Representative images of H&E staining of the mouse lung tumor burden. **g** and **h**. IHC of VEGF and HUR in lung metastatic lesions of mice. **i** and **j**. AOD of VEGF and HUR staining in lung metastatic lesions of mice. Quantitative data from three independent experiments are shown as the mean ± SD (error bars). *P < 0.05, **P < 0.01, ***P < 0.001 (Student’s t-test)

To investigate the metastatic potential of circSHKBP1 in vivo, we first stably transfected the abovementioned 5 cell lines with a luciferase plasmid and then injected these cells into nude mice via the tail vein. Half of the mice in the LV5-circSHKBP1 and LV5-NC groups were administrated bevacizumab twice a week. Three weeks after injection, lung metastasis was detected by in vivo bioluminescence imaging per week for 4 weeks. The results showed that circSHKBP1 knockdown dramatically decreased the number and size of lung metastatic lesions as detected by bioluminescence imaging and H&E staining (Fig. 7a-c and S5G). Moreover, IHC showed that circSHKBP1 knockdown resulted in obviously reduced HUR and VEGF staining in GC lung metastatic lesions (Fig. 7d). Overexpression of circSHKBP1 aggravated lung metastatic lesions, as shown by bioluminescence imaging and H&E staining and increased HUR and VEGF staining (Fig. 7e-h and S5H). The metastatic potential of circSHKBP1 was also inhibited by bevacizumab.

## Discussion

CircRNAs are a class of endogenous ncRNAs with abundant and stable expression. To date, numerous circRNAs have been identified from the human genome with the advent of next-generation sequencing. However, the biological functions of circRNAs in cancers are not well known. Using RNA-seq, we identified an oncogenic circRNA derived from the SHKBP1 gene, circSHKBP1, which was upregulated in GC tissues compared to matched normal tissues. CircSHKBP1 was abundant in serum exosomes and was similarly expressed in the serum and tumors of GC patients. Exosomal circSHKBP1 reducing after gastrectomy meant that GC tumors were the origin of circSHKBP1 in serum exosomes and exosomal circSHKBP1 indicated the existence of GC cells. Furthermore, overexpression of circSHKBP1 was associated with advanced TNM stage, vascular invasion and poor prognosis. These results suggest that circSHKBP1 is a stable biomarker for GC diagnosis and prognosis.

**Fig. 8:**
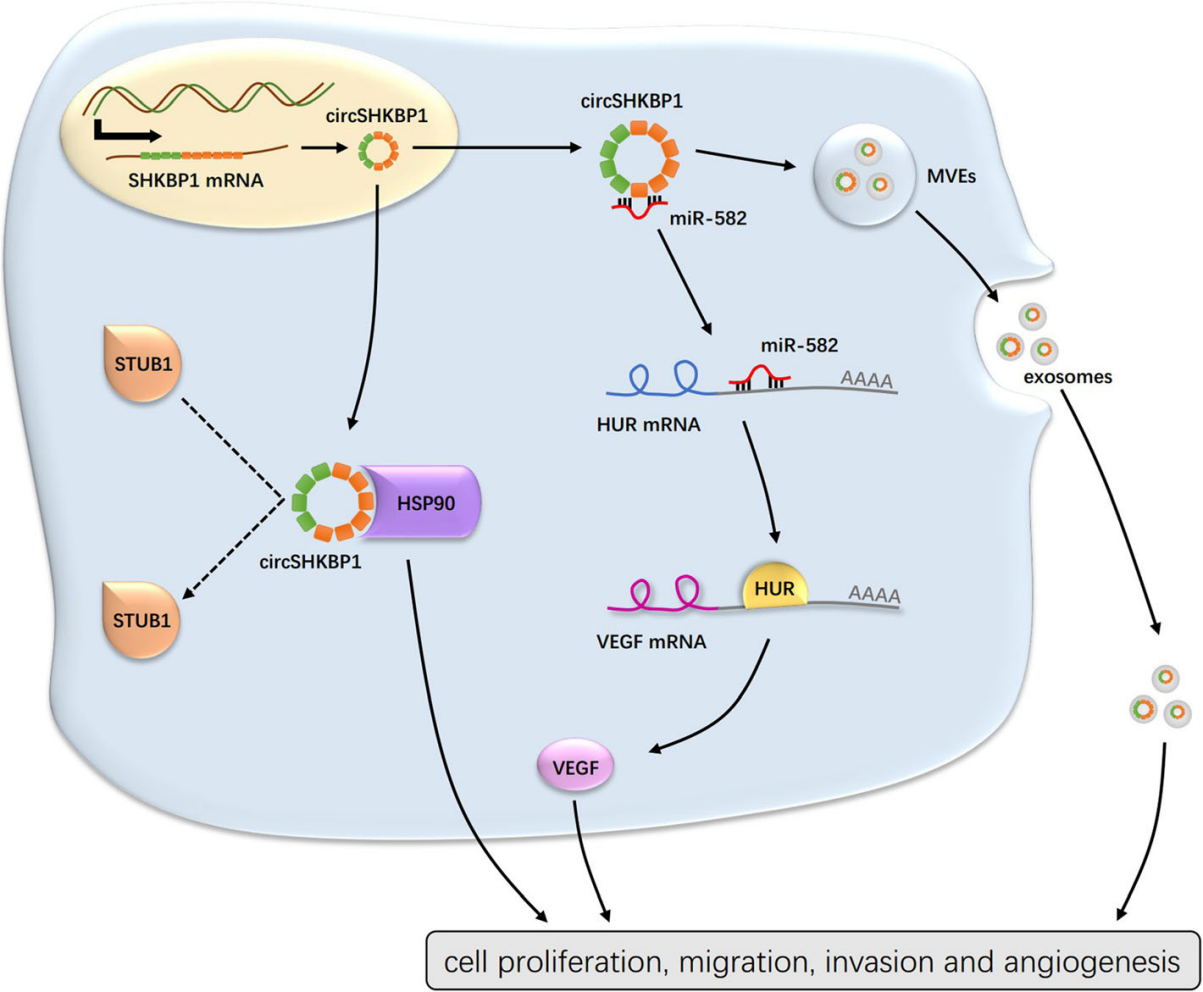
Schematic of the action of circSHKBP1 regulating gastric cancer progression

Studies have shown that circRNAs are differentially expressed between tumor and normal tissues, which means that they may play important roles in tumorigenesis. Experiments in vitro and in vivo have demonstrated the regulatory functions of circRNAs in cancer development by sponging miRNAs, decoying proteins, and affecting translation. In this study, we found that circSHKBP1 promoted GC development by sponging miR-582-3p and decoying HSP90. And ectopic circSHKBP1 delivered by exosomes into peripheral circulation affects distant GC cell growth, accelerating GC development. Intracellularly, circSHKBP1 binds to miR-582-3p and reduces the expression of miR-582-3p, which has been identified as a tumor suppressor in several kinds of solid tumors [33]. HUR, the downstream target of miR-582-3p, is expressed in an opposite pattern to miR-582-3p and is highly expressed in GC. HUR stabilizes the mRNA and leads to active translation of VEGF, enhancing angiogenesis and inducing tumor development. Moreover, circSHKBP1 slows down the degradation of oncoprotein HSP90 by competing with the binding site of ubiquitin ligase STUB1 (Fig. 8). In vivo, knockdown of circSHKBP1 suppresses the growth of subcutaneously implanted tumors and reduces pulmonary metastasis in nude mice. And overexpression of circSHKBP1 promotes both subcutaneously implanted tumors and pulmonary metastasis, which can be inhibited by administration of bevacizumab.

Although we have identified circSHKBP1 as a tumor promoter in GC and clarified its regulatory mechanism, there are several aspects to be improved. Firstly, it would be better to perform RNA-seq on serum exosomes of GC patients to acquire profiles of differentially expressed exosomal circRNAs between GC patients and healthy controls. We confirmed overexpression of exosomal circSHKBP1 by qRT-PCR instead of RNA-seq, which means that circSHKBP1 was not necessarily the one with the greatest difference in serum exosomes. However, we demonstrated that circSHKBP1 was highly abundant in exosomes and expressed similarly to intracellular circSHKBP1. Additionally, exosomes with high levels of circSHKBP1 increased GC cell growth and metastasis, suggesting that circSHKBP1 could be delivered by exosomes, participate in cell communication, and be a valid molecular target for liquid biopsy. Secondly, we only implemented treatment against VEGF and HSP90, verifying that the tumor-promoting action of circSHKBP1 could be blocked by an anti-VEGF antibody and HSP90 inhibitor. As we have identified circSHKBP1 as an oncogene, the therapeutic effect of anti-circSHKBP1 treatment should be evaluated for GC molecular targeted therapy. Importantly, it is clear that silencing or overexpressing circSHKBP1 in subcutaneous tumors did not impair the basic physiological functions of nude mice.

In conclusion, we demonstrate that circSHKBP1 is upregulated in GC patients and is associated with advanced TNM stage, vascular invasion and poor prognosis. Liquid biopsy of serum exosomes targeting circSHKBP1 can help diagnose and predict the prognosis of GC. CircSHKBP1 promotes GC progression by sponging miR-582-3p to upregulate HUR and VEGF as well as decoying HSP90 by competing with STUB1. Consequently, circSHKBP1 is considered a promising biomarker for GC diagnosis and prognosis, and it is a potential therapeutic target for GC treatment.

## Supplementary information

**Additional file 1: Figure S1.** Alu elements of circSHKBP1 and identification of serum exosomes. (A). Alu elements in the flanking sequence blasted by NCBI. (B) Expression of SHKBP1 in GC tumors (*n* = 408) and normal tissues (*n* = 211) obtained from TCGA database. (C) Expression of SHKBP1 in paired GC tumors and normal tissues (*n* = 72). (D) Levels of circSHKBP1 and SHKBP1 mRNA in BGC823 cells treated with RNase R, as determined by RT-PCR (top panel) and qRT-PCR (bottom panel). (E). Levels of circSHKBP1 and SHKBP1 mRNA in BGC823 cells treated with actinomycin D at the indicated time points detected by qRT-PCR. (F). Serum exosomes identified by TEM and western blot.

**Additional file 2: Figure S2.** CircSHKBP1 promotes GC cell proliferation, migration and invasion in vitro. (A). Level of SHKBP1 mRNA in BGC823, HGC27, AGS, MGC803, and GES1 cells, as determined by qRT-PCR (normalized to GES1 levels). (B and C). Level of circSHKBP1 and SHKBP1 mRNA in BGC823 and HGC27 cells transfected with circSHKBP1 siRNAs or control, as determined by qRT-PCR. (D and E). Levels of circSHKBP1 and SHKBP1 mRNA in BGC823 and HGC27 cells transfected with control vector or circSHKBP1 plasmid, as determined by qRT-PCR. (F). Assessment of the proliferation of HGC27 cells transfected with control or circSHKBP1 siRNAs by CCK8 assay. (G and J). Assessment of the proliferation of HGC27 cells transfected with control or circSHKBP1 siRNAs by EdU assay. (H) Assessment of the proliferation of HGC27 cells transfected with control vector or circSHKBP1 plasmid by CCK8 assay. (I and M). Assessment of the proliferation of HGC27 cells transfected with control vector or circSHKBP1 plasmid by EdU assay. (K and L). Assessment of the migration and invasion of HGC27 cells transfected with control or circSHKBP1 siRNAs by Transwell assay. (N and O). Assessment of the migration and invasion of HGC27 cells transfected with control vector or circSHKBP1 plasmid by Transwell assay. (P). Level of exosomal circSHKBP1 in BGC823 and HGC27 cells transfected with control vector or circSHKBP1 plasmid, as determined by qRT-PCR. (Q). Level of exosomal circSHKBP1 in BGC823 and HGC27 cells transfected with circSHKBP1 siRNAs or control, as determined by qRT-PCR. (R) Total amount of medium exosomes of BGC823 cells transfected with control vector (right panel) or circSHKBP1 plasmid (left panel), as determined by NTA. Quantitative data from three independent experiments are shown as the mean ± SD (error bars). *P < 0.05, **P < 0.01, ***P < 0.001 (Student’s t-test).

**Additional file 3: Figure S3.** CircSHKBP1 serves as a sponge of miR-582-3p. (A). Dual luciferase reporter assay was used to detect the relative luciferase activity (firefly/renilla) in HEK-293 T cells cotransfected with miR-665 mimics and pmirGLO-circSHKBP1-miR-665 WT/MUT. (B). Level of circSHKBP1 pulled down by biotin-labeled miR-582 or control probe. (C and D). Expression of miR-582 and circSHKBP1 in BGC823 and HGC27 cells transfected with control vector, circSHKBP1 plasmid, or miR-582 mimic or cotransfected with miR-582 mimic and circSHKBP1 plasmid, as determined by qRT-PCR. (E). Assessment of the proliferation of HGC27 cells transfected with control vector, circSHKBP1 plasmid, or miR-582 mimic or cotransfected with miR-582 mimic and circSHKBP1 plasmid by CCK8 assay. (F and G). Assessment of the migration and invasion of HGC27 cells transfected with control vector, circSHKBP1 plasmid, or miR-582 mimic or cotransfected with miR-582 mimic and circSHKBP1 plasmid by Transwell assay. Quantitative data from three independent experiments are shown as the mean ± SD (error bars). *P < 0.05, **P < 0.01, ***P < 0.001 (Student’s t-test).

**Additional file 4: Figure S4.** CircSHKBP1 promotes VEGF translation by upregulating HUR. (A and B). Expression of HUR and EIF2S1 in GC tumors (*n* = 408) and normal tissues (*n* = 211) obtained from TCGA database. (C and D). Survival analysis of HUR (*n* = 876, median survival (months): low expression vs. high expression = 25 vs. 23, *P* = 7.4 × 10^− 5^) and EIF2S1 (*n* = 876, *P* = 0.41) for GC. The median expression levels of HUR and EIF2S1 were used as the cutoff value. Log-rank tests were used to determine statistical significance. (E). Western blot showing the expression level of EIF2S1 in BGC823 cells transfected with control vector, circSHKBP1 plasmid, miR-582 mimic, or miR-582 inhibitor or cotransfected with miR-582 mimic and circSHKBP1 plasmid (tubulin as an internal control). (F). Western blot showing the expression level of VEGF and HUR in HGC27 cells transfected with control mimic, miR-582 mimic, control inhibitor or miR-582 inhibitor; expression level of VEGF and HUR in HGC27 cells transfected with control vector or circSHKBP1 plasmid or cotransfected with miR-582 mimic and circSHKBP1 plasmid; expression level of VEGF and HUR in HGC27 cells transfected with control or circSHKBP1 siRNA or cotransfected with miR-582 inhibitor and circSHKBP1 siRNA (tubulin as an internal control). (H). Western blot showing the expression level of VEGF in HGC27 cells transfected with control vector or circSHKBP1 plasmid treated with bevacizumab at different concentrations. (I and J). Assessment of the migration of HGC27 cells transfected with control vector or circSHKBP1 plasmid and treated with bevacizumab at different concentrations by Transwell assay. (K). Assessment of the proliferation of HGC27 cells transfected with control vector or circSHKBP1 plasmid and treated with bevacizumab at different concentrations by CCK8 assay. (L). Inhibition rate of bevacizumab of the proliferation and migration of HGC27 cells transfected with control vector or circSHKBP1 plasmid. Quantitative data from three independent experiments are shown as the mean ± SD (error bars). *P < 0.05, **P < 0.01, ***P < 0.001 (Student’s t-test).

**Additional file 5: Figure S5.** Efficiency of stably transfected cells, body weight and xenograft tumor volume monitored in animal models. (A). Level of circSHKBP1 in BGC823 LV3-NC, LV3-sh1 and LV3-sh2 cells determined by qRT-PCR. (B) Level of circSHKBP1 in BGC823 LV5-NC and LV5-circSHKBP1 cells determined by qRT-PCR. (C and D) Body weight of mice with xenograft tumors monitored every 3 days. (E and F) Xenograft tumor volume of mice monitored every 3 days. (G and H) Body weight of mice receiving tail vein injection monitored every week. Quantitative data from three independent experiments are shown as the mean ± SD (error bars). *P < 0.05, **P < 0.01, ***P < 0.001 (Student’s t-test).

**Additional file 6.**

**Additional file 7.**

**Additional file 8.**

## Data Availability

The data supporting the conclusions of this article are presented within the article and its additional files.

## Abbreviations

CircRNAs: Circular RNAs
GC: Gastric cancer
qRT-PCR: Quantitative real-time PCR
ncRNAs: Noncoding RNAs
miRNAs: MicroRNAs
mRNA: Messenger RNA
Let-7: Lethal-7
HUR: Human antigen R
RBPs: RNA binding proteins
AREs: AU-rich elements
VEGF: Vascular endothelial growth factor
HSP90: Heat shock protein 90
FBS: Fetal bovine serum
CHX: Cycloheximide
FC: Fold change
BEV: Bevacizumab
RNA-seq: RNA sequencing
FISH: RNA fluorescence in situ hybridization
CCK8: Cell counting kit-8
EdU: 5-Ethynyl-2′-deoxyuridine
IHC: Immunohistochemistry
IF: Immunofluorescence
IP: Immunoprecipitation
RIP: RNA-protein immunoprecipitation
ISH: RNA in situ hybridization
BLI: Bioluminescence imaging
3’UTR: 3′ untranslated region
ELISA: Enzyme-linked immunosorbent assay
TEM: Transmission electron microscope
