# Supplementary figures and images for: Exosomal circSHKBP1 promotes gastric cancer progression via regulating the miR-582-3p/HUR/VEGF axis and suppressing HSP90 degradation
Source: Mol Cancer. 2020 Jun 29;19:112. doi: 10.1186/s12943-020-01208-3 (PMC7322843; doi:10.1186/s12943-020-01208-3)

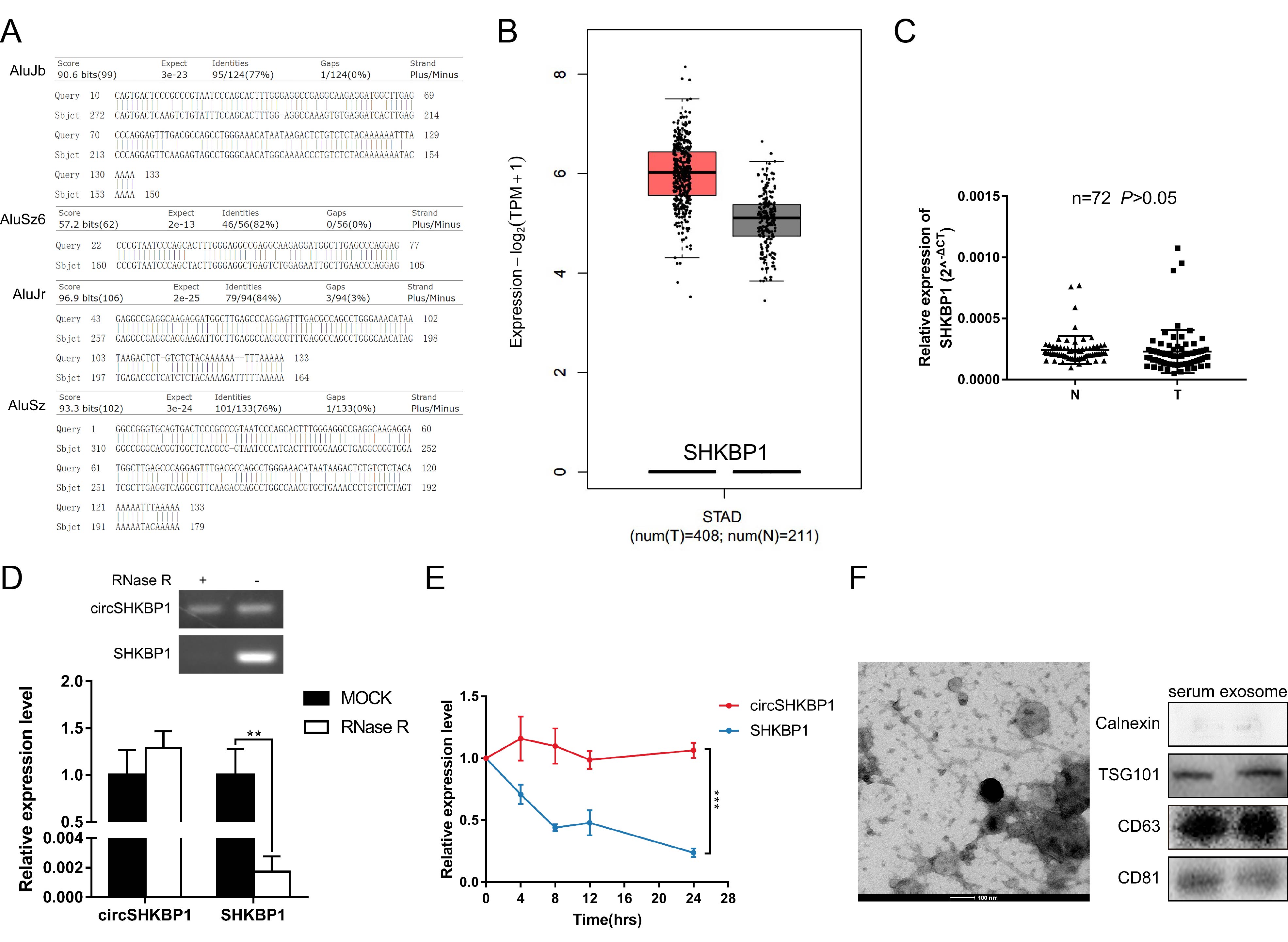

Supplement: Supplementary file 1 — Additional file 1: Figure S1. Alu elements of circSHKBP1 and identification of serum exosomes. (A). Alu elements in the flanking sequence blasted by NCBI. (B) Expression of SHKBP1 in GC tumors (n = 408) and normal tissues (n = 211) obtained from TCGA database. (C) Expression of SHKBP1 in paired GC tumors and normal tissues (n = 72). (D) Levels of circSHKBP1 and SHKBP1 mRNA in BGC823 cells treated with RNase R, as determined by RT-PCR (top panel) and qRT-PCR (bottom panel). (E). Levels of circSHKBP1 and SHKBP1 mRNA in BGC823 cells treated with actinomycin D at the indicated time points detected by qRT-PCR. (F). Serum exosomes identified by TEM and western blot. [file 12943_2020_1208_MOESM1_ESM.jpg]

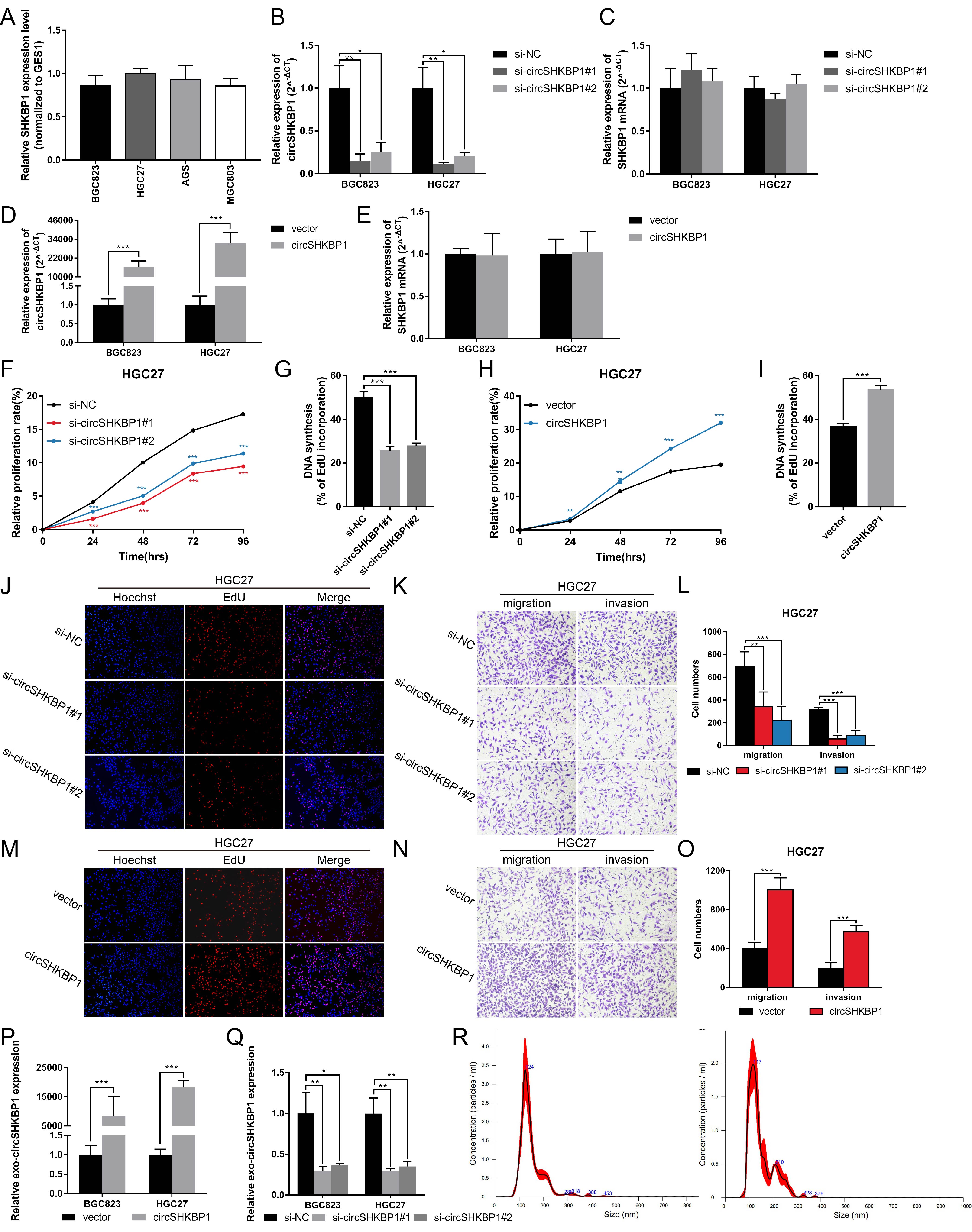

Supplement: Supplementary file 2 — Additional file 2: Figure S2. CircSHKBP1 promotes GC cell proliferation, migration and invasion in vitro. (A). Level of SHKBP1 mRNA in BGC823, HGC27, AGS, MGC803, and GES1 cells, as determined by qRT-PCR (normalized to GES1 levels). (B and C). Level of circSHKBP1 and SHKBP1 mRNA in BGC823 and HGC27 cells transfected with circSHKBP1 siRNAs or control, as determined by qRT-PCR. (D and E). Levels of circSHKBP1 and SHKBP1 mRNA in BGC823 and HGC27 cells transfected with control vector or circSHKBP1 plasmid, as determined by qRT-PCR. (F). Assessment of the proliferation of HGC27 cells transfected with control or circSHKBP1 siRNAs by CCK8 assay. (G and J). Assessment of the proliferation of HGC27 cells transfected with control or circSHKBP1 siRNAs by EdU assay. (H) Assessment of the proliferation of HGC27 cells transfected with control vector or circSHKBP1 plasmid by CCK8 assay. (I and M). Assessment of the proliferation of HGC27 cells transfected with control vector or circSHKBP1 plasmid by EdU assay. (K and L). Assessment of the migration and invasion of HGC27 cells transfected with control or circSHKBP1 siRNAs by Transwell assay. (N and O). Assessment of the migration and invasion of HGC27 cells transfected with control vector or circSHKBP1 plasmid by Transwell assay. (P). Level of exosomal circSHKBP1 in BGC823 and HGC27 cells transfected with control vector or circSHKBP1 plasmid, as determined by qRT-PCR. (Q). Level of exosomal circSHKBP1 in BGC823 and HGC27 cells transfected with circSHKBP1 siRNAs or control, as determined by qRT-PCR. (R) Total amount of medium exosomes of BGC823 cells transfected with control vector (right panel) or circSHKBP1 plasmid (left panel), as determined by NTA. Quantitative data from three independent experiments are shown as the mean ± SD (error bars). *P < 0.05, **P < 0.01, ***P < 0.001 (Student’s t-test). [file 12943_2020_1208_MOESM2_ESM.jpg]

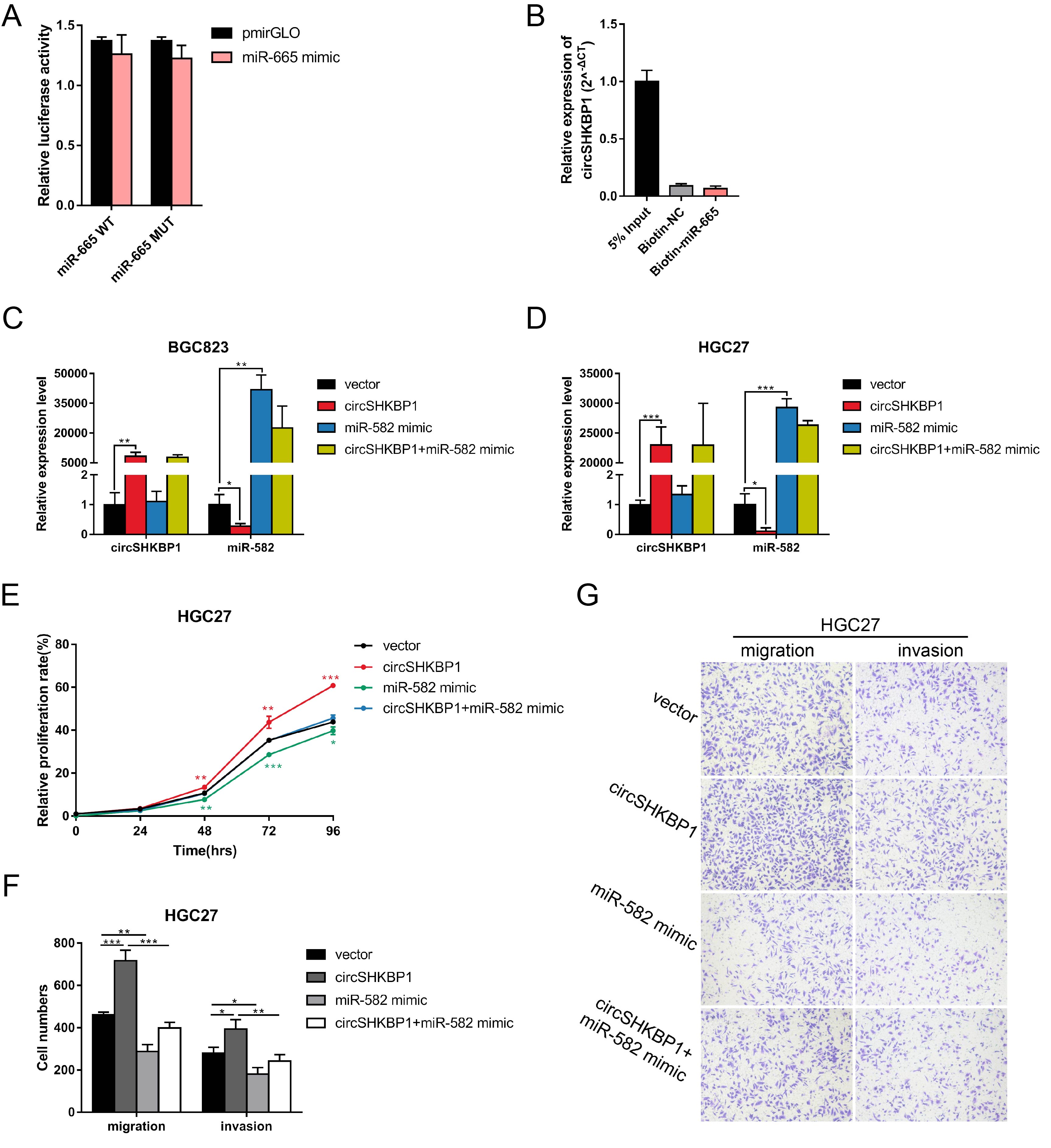

Supplement: Supplementary file 3 — Additional file 3: Figure S3. CircSHKBP1 serves as a sponge of miR-582-3p. (A). Dual luciferase reporter assay was used to detect the relative luciferase activity (firefly/renilla) in HEK-293 T cells cotransfected with miR-665 mimics and pmirGLO-circSHKBP1-miR-665 WT/MUT. (B). Level of circSHKBP1 pulled down by biotin-labeled miR-582 or control probe. (C and D). Expression of miR-582 and circSHKBP1 in BGC823 and HGC27 cells transfected with control vector, circSHKBP1 plasmid, or miR-582 mimic or cotransfected with miR-582 mimic and circSHKBP1 plasmid, as determined by qRT-PCR. (E). Assessment of the proliferation of HGC27 cells transfected with control vector, circSHKBP1 plasmid, or miR-582 mimic or cotransfected with miR-582 mimic and circSHKBP1 plasmid by CCK8 assay. (F and G). Assessment of the migration and invasion of HGC27 cells transfected with control vector, circSHKBP1 plasmid, or miR-582 mimic or cotransfected with miR-582 mimic and circSHKBP1 plasmid by Transwell assay. Quantitative data from three independent experiments are shown as the mean ± SD (error bars). *P < 0.05, **P < 0.01, ***P < 0.001 (Student’s t-test). [file 12943_2020_1208_MOESM3_ESM.jpg]

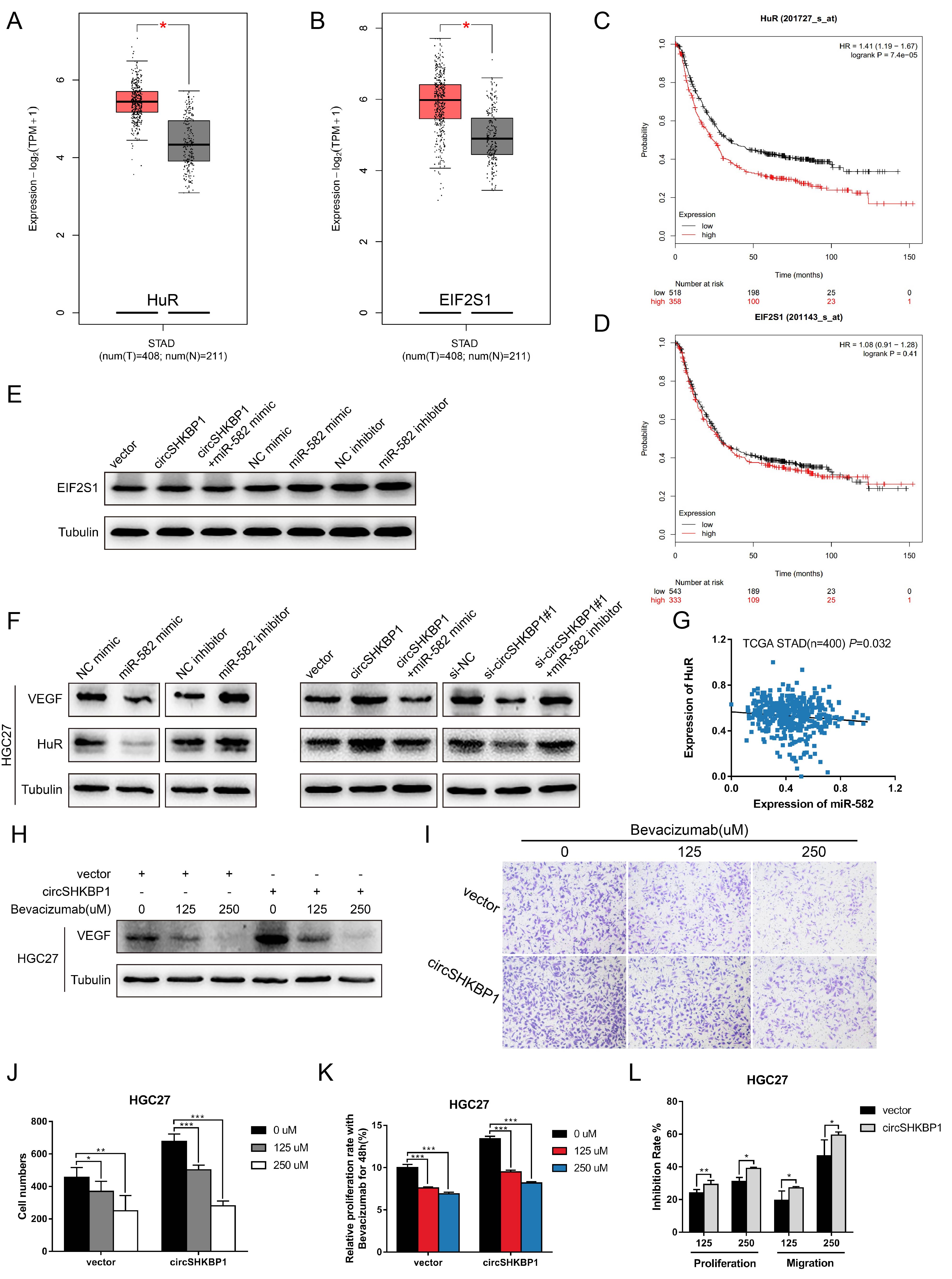

Supplement: Supplementary file 4 — Additional file 4: Figure S4. CircSHKBP1 promotes VEGF translation by upregulating HUR. (A and B). Expression of HUR and EIF2S1 in GC tumors (n = 408) and normal tissues (n = 211) obtained from TCGA database. (C and D). Survival analysis of HUR (n = 876, median survival (months): low expression vs. high expression = 25 vs. 23, P = 7.4 × 10− 5) and EIF2S1 (n = 876, P = 0.41) for GC. The median expression levels of HUR and EIF2S1 were used as the cutoff value. Log-rank tests were used to determine statistical significance. (E). Western blot showing the expression level of EIF2S1 in BGC823 cells transfected with control vector, circSHKBP1 plasmid, miR-582 mimic, or miR-582 inhibitor or cotransfected with miR-582 mimic and circSHKBP1 plasmid (tubulin as an internal control). (F). Western blot showing the expression level of VEGF and HUR in HGC27 cells transfected with control mimic, miR-582 mimic, control inhibitor or miR-582 inhibitor; expression level of VEGF and HUR in HGC27 cells transfected with control vector or circSHKBP1 plasmid or cotransfected with miR-582 mimic and circSHKBP1 plasmid; expression level of VEGF and HUR in HGC27 cells transfected with control or circSHKBP1 siRNA or cotransfected with miR-582 inhibitor and circSHKBP1 siRNA (tubulin as an internal control). (H). Western blot showing the expression level of VEGF in HGC27 cells transfected with control vector or circSHKBP1 plasmid treated with bevacizumab at different concentrations. (I and J). Assessment of the migration of HGC27 cells transfected with control vector or circSHKBP1 plasmid and treated with bevacizumab at different concentrations by Transwell assay. (K). Assessment of the proliferation of HGC27 cells transfected with control vector or circSHKBP1 plasmid and treated with bevacizumab at different concentrations by CCK8 assay. (L). Inhibition rate of bevacizumab of the proliferation and migration of HGC27 cells transfected with control vector or circSHKBP1 plasmid. Quantitative data fro [file 12943_2020_1208_MOESM4_ESM.jpg]

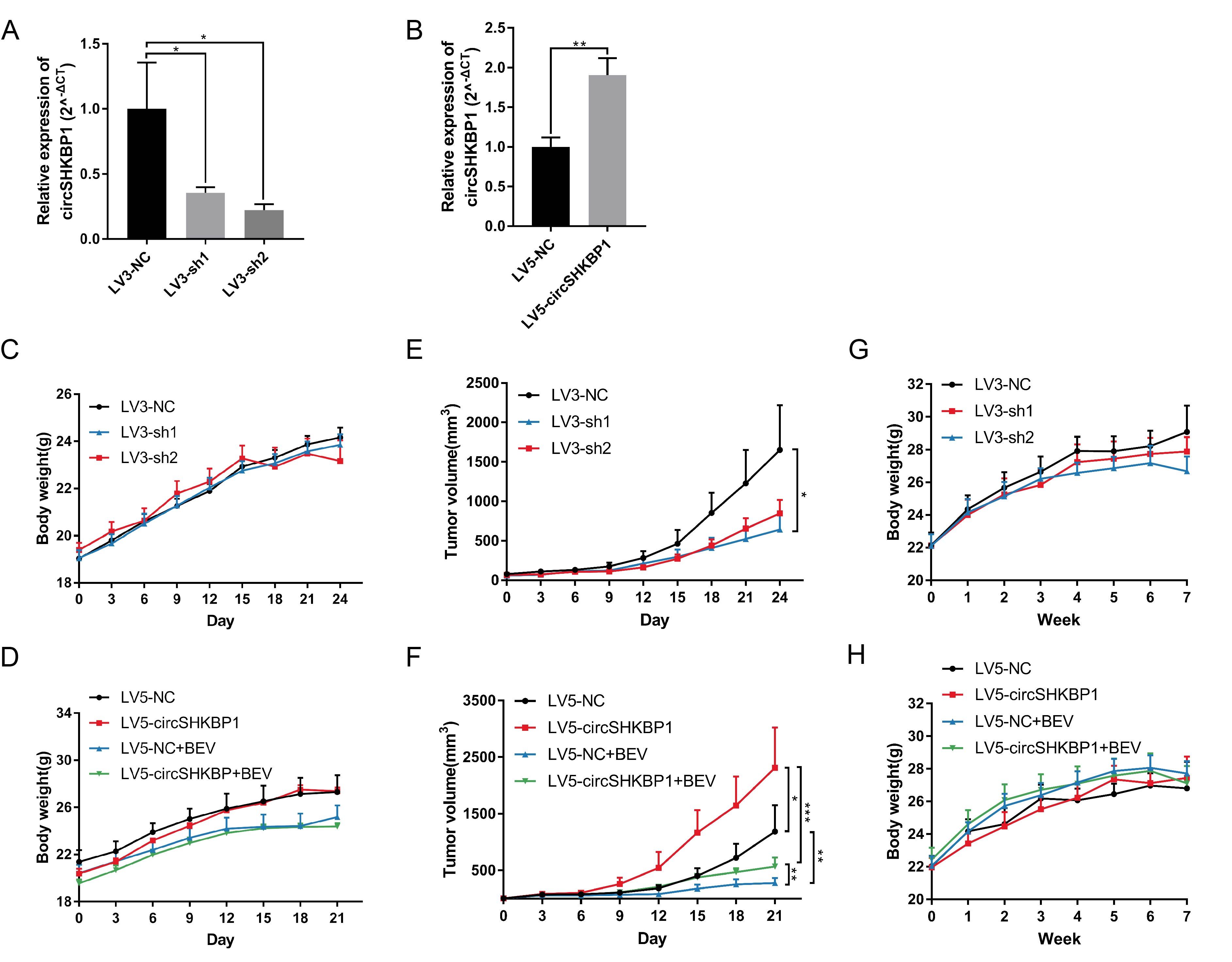

Supplement: Supplementary file 5 — Additional file 5: Figure S5. Efficiency of stably transfected cells, body weight and xenograft tumor volume monitored in animal models. (A). Level of circSHKBP1 in BGC823 LV3-NC, LV3-sh1 and LV3-sh2 cells determined by qRT-PCR. (B) Level of circSHKBP1 in BGC823 LV5-NC and LV5-circSHKBP1 cells determined by qRT-PCR. (C and D) Body weight of mice with xenograft tumors monitored every 3 days. (E and F) Xenograft tumor volume of mice monitored every 3 days. (G and H) Body weight of mice receiving tail vein injection monitored every week. Quantitative data from three independent experiments are shown as the mean ± SD (error bars). *P < 0.05, **P < 0.01, ***P < 0.001 (Student’s t-test). [file 12943_2020_1208_MOESM5_ESM.jpg]
